# Artificial Intelligence of Things (AIoT) Enabled Virtual Shop Applications Using Self‐Powered Sensor Enhanced Soft Robotic Manipulator

**DOI:** 10.1002/advs.202100230

**Published:** 2021-05-26

**Authors:** Zhongda Sun, Minglu Zhu, Zixuan Zhang, Zhaocong Chen, Qiongfeng Shi, Xuechuan Shan, Raye Chen Hua Yeow, Chengkuo Lee

**Affiliations:** ^1^ Department of Electrical & Computer Engineering National University of Singapore 4 Engineering Drive 3 Singapore 117576 Singapore; ^2^ Institute of Manufacturing Technology and National University of Singapore (SIMTech‐NUS) Joint Lab on Large‐Area Flexible Hybrid Electronics National University of Singapore 4 Engineering Drive 3 Singapore 117576 Singapore; ^3^ Center for Intelligent Sensors and MEMS (CISM) National University of Singapore 5 Engineering Drive 1 Singapore 117608 Singapore; ^4^ National University of Singapore Suzhou Research Institute (NUSRI) Suzhou Industrial Park Suzhou 215123 China; ^5^ Printed Intelligent Device Group Singapore Institute of Manufacturing Technology (SIMTech) Agency for Science Technology and Research (A*STAR) Singapore 637662 Singapore; ^6^ Department of Biomedical Engineering National University of Singapore #04‐08, Engineering Block 4, 4 Engineering Drive 3 Singapore 117583 Singapore

**Keywords:** artificial intelligence, object recognition, soft manipulator, triboelectric, virtual/augmented reality

## Abstract

Rapid advancements of artificial intelligence of things (AIoT) technology pave the way for developing a digital‐twin‐based remote interactive system for advanced robotic‐enabled industrial automation and virtual shopping. The embedded multifunctional perception system is urged for better interaction and user experience. To realize such a system, a smart soft robotic manipulator is presented that consists of a triboelectric nanogenerator tactile (T‐TENG) and length (L‐TENG) sensor, as well as a poly(vinylidene fluoride) (PVDF) pyroelectric temperature sensor. With the aid of machine learning (ML) for data processing, the fusion of the T‐TENG and L‐TENG sensors can realize the automatic recognition of the grasped objects with the accuracy of 97.143% for 28 different shapes of objects, while the temperature distribution can also be obtained through the pyroelectric sensor. By leveraging the IoT and artificial intelligence (AI) analytics, a digital‐twin‐based virtual shop is successfully implemented to provide the users with real‐time feedback about the details of the product. In general, by offering a more immersive experience in human–machine interactions, the proposed remote interactive system shows the great potential of being the advanced human–machine interface for the applications of the unmanned working space.

## Graphical abstract

A smart soft robotic manipulator is developed with a self‐powered multifunctional sensory system for simultaneously deformation, tactile, and temperature perception. With machine learning analysis, automatic recognition of grasped objects can be realized with high accuracy. By leveraging the artificial intelligence of things (AIoT) technology, a digital‐twin‐based virtual shop is successfully implemented to provide users with a more immersive shopping experience.

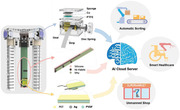

## 1 Introduction

Digital twin refers to a digital replica that can provide real‐time monitoring or maintenance optimization of physical systems in the manufacturing, healthcare, and automotive industry.^[^ ^1^, ^2^ ^]^ With the gradual rollout of the 5G and Internet of Things (IoT) technology across the world, the digital twin will be much easier to be achieved. Based on the information from the distributed sensory network, one of the key applications is to inspect the physical objects with the up‐to‐date status, including both static properties, i.e., inherent features of the physical objects such as shape, size, color, etc., and dynamic properties, i.e., the real‐time position, movement, gesture, and motion of objects that will change over time, etc.^[^ ^3^ ^]^ This type of cyber‐physical system enables the advanced interaction virtually and remotely,^[^ ^4^ ^]^ showing the potential in realizing real‐time parallel control in unmanned working spaces. Currently, the global pandemic of the coronavirus disease 2019 (COVID‐19) has made staying at home a normal life. The digital‐twin‐based remote interactive system may bring great convenience in various scenarios, such as online shopping and unmanned factory. Specifically, online shopping has become an indispensable part of our daily life in the information age.^[^ ^5^ ^]^ The online virtual shop system will not only provide immersive experience and more details about the products in the augmented reality (AR) or virtual reality (VR) spaces, but also enables the intelligent robots to perform the joint operations and to provide real‐time feedback sensation in real space. For establishing such a virtual shop system for shop floor management and screening of merchandise, except for the conventional methods through computer mouse, touchscreen, or video‐based observation, many works have been done recently to develop wearable manipulators^[^ ^6^, ^7^, ^8^, ^9^ ^]^ that provide immersive experience to users with the sensory and haptic feedback functions, such as gesture control, direct projection of body motions, and haptic stimulators. While for the anthropomorphic robots in real space, the realization of the human perception system is essential to provide feedback information precisely. Compared with the rigid robotic manipulators widely used in various industries, soft robots^[^ ^10^, ^11^, ^12^, ^13^, ^14^, ^15^, ^16^, ^17^, ^18^, ^19^, ^20^, ^21^ ^]^ made by flexible materials, e.g., thermoplastic polyurethanes (TPU), are more suitable to fabricate the humanoid robotic finger due to the flexibility, lightweight, multidegree of freedom, and excellent conformability, etc.^[^ ^22^, ^23^, ^24^, ^25^, ^26^, ^27^, ^28^, ^29^, ^30^, ^31^, ^32^, ^33^, ^34^, ^35^, ^36^, ^37^ ^]^ Generally, the low‐cost solution of a soft robotic system with marginal sensing functions is desired for the massive deployment of the humanoid robotics.

Currently, many works have used the camera as the sensing unit to help robots to perceive the external world.^[^ ^38^, ^39^, ^40^, ^41^ ^]^ However, this strategy has limitations in the dark environment of many unmanned spaces due to the low visibility and poor image quality. In this case, the infra‐red (IR) camera is widely adopted as the sensing method for the dark environment, which provides not only the temperature information but also the shape‐related information of the objects. However, most of the recent IR‐images related works are focus on the object detection in the dark,^[^ ^42^, ^43^ ^]^, e.g., vehicle and pedestrian detection, where the object recognition is a two‐class classification service for target detection, which means only target and nontarget recognition are required. The scenario of multiobject classification, especially indoor object recognition, has not been given enough attention due to the relatively lower resolution and fewer object features that can be captured compared with the visible light‐based camera. Under this circumstance, an embedded sensory system that is highly compatible with the robots for working assistance in a dark environment can be a complementary solution for the dark space applications in addition to the technology using IR camera‐based indoor object recognition, with the advantages of providing additional features other than that based on visual to enhance the recognition ability, as well as serving as a low‐cost solution, i.e., saving bandwidth and computing resources, due to the much lower data complexity compared with that of visual images.

By considering the large deformability of the soft robots, flexible tactile and strain sensors^[^ ^44^, ^45^, ^46^, ^47^, ^48^, ^49^, ^50^, ^51^, ^52^, ^53^, ^54^, ^55^, ^56^, ^57^, ^58^, ^59^, ^60^, ^61^, ^62^, ^63^, ^64^ ^]^ have been developed frequently due to the better compatibility compared to the conventional rigid sensors, i.e., potentiometer and encoder. For instance, Goldoni et al. have proposed a nanostructured resistive strain sensor based on a carbon nanomaterial for monitoring the motions of a robotic segment.^[^ ^44^ ^]^ Bai et al. have proposed a silica‐based distributed fiber‐optical sensor that can measure the mechanical deformation of stretching, bending, or pressing for soft robotics.^[^ ^45^ ^]^ In summary, current common mechanical stimulus sensing approaches for soft robots include conductive nanocomposites,^[^ ^65^, ^66^ ^]^ photodetection,^[^ ^67^, ^68^ ^]^ and electromagnetic effect.^[^ ^69^ ^]^ Besides, the temperature sensing ability is also important for the robot perception. Recent flexible solutions on accurate 2D temperature mapping show the possibility of realizing a more biomimetic temperature sensing system with high sensitivity and flexibility. Gong et al. have developed a graphene nanoribbon‐based flexible temperature sensor with high thermosensitivity that can be used for body temperature monitoring, human touch identification, and 5 × 5 array temperature mapping.^[^ ^70^ ^]^ Shin et al. have reported a negative temperature coefficient thermistor‐based artificial skin, which can be attached to the robotic hand in array to simulate the network of thermoreceptors densely distributed on the human skin with excellent spatial resolution.^[^ ^71^ ^]^ Moreover, an emerging sensor fusion concept that integrates sensors capable of detecting various parameters, i.e., temperature, tactile, strain, etc., is also important in developing the multifunctional sensory system of anthropomorphic robotic fingers. Li et al. have reported a quadruple tactile sensor capable of pressure sensing, material thermal conductivity sensing, and bimodal temperature sensing, so that the shape, material, and texture of objects can be perceived through the fusion of multiple sensing information.^[^ ^72^ ^]^ Wan et al. have proposed a bimodal artificial sensory neuron based on ionic/electronic hybrid neuromorphic electronics to implement the visual‐haptic fusion, providing multidimensional spatial information for the robotic hand.^[^ ^73^ ^]^ However, many of these mentioned methods are still confronted with challenges in cost effectiveness, energy consumption, compatibility of materials and process. Based on this, the flexible self‐powered approaches, including triboelectricity^[^ ^74^, ^75^ ^]^ and piezoelectricity^[^ ^76^ ^]^ for stimulus detection, and thermoelectricity^[^ ^77^ ^]^ and pyroelectricity^[^ ^78^ ^]^ for temperature sensing, will have great advantages in realizing long‐term sustainable IoT intelligent systems because these sensors can generate electrical signals without external electrical bias, i.e., zero‐power consumption at the sensor itself, and are made with low‐cost fabrication technology. These advantages are indispensable for enabling massive sensor nodes to collect multimodal sensory information aiming at the future ubiquitous IoT framework.

Triboelectric nanogenerator (TENG) refers to a device or a mechanism using the coupling effect of triboelectrification and electrostatic induction to generate electrical charges. This mechanism has been frequently studied as an emerging energy harvesting technology that can efficiently scavenge the mechanical energy.^[^ ^79^, ^80^ ^]^ Compared with flexible piezoelectric‐based sensors, TENGs possess the advantages of low cost, high flexibility brought by the wide options of materials, making it being widely explored as self‐powered wearable sensors for strain, tactile, and gesture sensing.^[^ ^7^, ^81^, ^82^, ^83^, ^84^, ^85^, ^86^, ^87^, ^88^, ^89^, ^90^, ^91^, ^92^, ^93^, ^94^, ^95^ ^]^ Meanwhile, pyroelectric‐based sensors with the self‐generated electrical signals when encountered with temperature changes,^[^ ^96^, ^97^, ^98^, ^99^, ^100^ ^]^ can be utilized as self‐powered temperature sensing units. With the increasing demand of flexibility for the wearable scenario, polymer pyroelectric materials, such as poly(vinylidene fluoride) (PVDF), polyvinylidene fluoride‐trifluoro ethylene (P(VDF‐TrFE)), have been widely investigated. Compared with the thermoelectric sensor whose performance is limited by the temperature gradient,^[^ ^77^ ^]^ pyroelectric‐based self‐powered temperature sensor capable of converting temperature change over time into electrical potential without strict working conditions shows great applicability in the wearable and robotic fields.^[^ ^101^, ^102^ ^]^ Song et al. developed a BaTiO3‐based pyro‐piezoelectric sensor system, in which pyroelectricity is applied to sense real‐time temperature variations induced by a finger.^[^ ^103^ ^]^ Wang et al. successfully utilized a PVDF layer as the actuation, as well as the temperature sensing unit of a light‐driven robot for integrated perception and motility.^[^ ^78^ ^]^

In terms of the advanced data interpretation, the new era of ML from AI paves the way for strengthening the functionalities of sensors with AI‐enhanced data analytic.^[^ ^104^, ^105^, ^106^ ^]^ Together with the AIoT network, more comprehensive sensory information can be extracted for more diversified applications in the wearable area. Kim et al. proposed a novel electronic skin sensor attached to the human wrist, which can real‐time decode the complex motion of five fingers with a deep neural network‐enabled rapid situation learning.^[^ ^107^ ^]^ Recently, several works have demonstrated the triboelectric‐based wearable sensors with AIoT technology for advanced human–machine interaction. Zhu et al. developed a glove with 16 triboelectric tactile sensors to realize grasped object recognition with high accuracy of 96%.^[^ ^7^ ^]^ Wen et al. successfully used ten triboelectric textile sensors for gesture identification with an accuracy of 95.23%.^[^ ^92^ ^]^ The subtle features hidden in the triboelectric waveform, including contact sequence, collision vibration, etc., can effectively enhance the perceptual capability of the integrated intelligent system.^[^ ^108^ ^]^ Compared with the data glove with the vast amount of sensor nodes,^[^ ^109^ ^]^ the minimalistic designs also show the comparable performance with the aid of ML analytics.

Similar to the aforementioned wearable sensors, TENG‐based flexible sensors are also utilized to realize the monitoring of the external stimulus and the self‐deformation for establishing the intelligent perception of the soft robotic system. Chen et al. integrated TENG sensors inside the chamber of a soft actuator for continuous bending measurement and feedback control.^[^ ^110^ ^]^ Lai et al. proposed triboelectric robotic skins for the active sensing of proximity, contact and pressure.^[^ ^111^ ^]^ Zhu et al. used 3D printing technology to directly print a soft robotic finger with a triboelectric curvature sensor.^[^ ^112^ ^]^ However, for realizing more complex functions, e.g., objects recognition, to enhance the intelligence of anthropomorphic robots in virtual shop application, the multimodal sensing data generated from different sensors are needed to be further interpreted and integrated with ML technology, especially for the concerns of identifying different objects with similar shapes, or the same objects with different grasping direction.^[^ ^72^, ^113^ ^]^ In addition, the pyroelectric‐based temperature perception is also important for soft robots to enhance the intelligence of object recognition, such as identifying the cold or hot drink. Overall, as an instance of the virtual shop, an advanced interactive system with both the intelligent soft manipulator with the multifunctional sensory system, and the immersive virtual interface which offers comprehensive information of the products in real space, will greatly improve users' experience.

**Figure 1:**
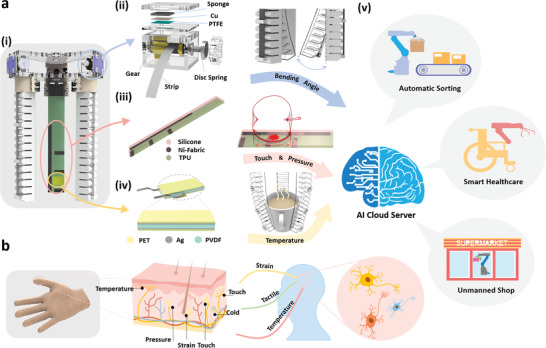
a‐i) The configuration of the sensor‐integrated smart manipulator. The structure and functionality of a‐ii) the L‐TENG sensor, a‐iii) the T‐TENG sensor and a‐iv) the PVDF temperature sensor. a‐v) Schematic diagram of diversified applications that can be enabled by the developed smart manipulator system. b) The strain, tactile, and temperature sensing function enabled by various types of receptors in human skin under biological neural network, corresponding to the three kinds of sensory information of bending angle, touch & pressure and temperature achieved by the L‐TENG, T‐TENG, and PVDF sensor respectively.

Herein, we integrate a soft‐robotic manipulator with an L‐TENG sensor (**Figure** **1**a‐ii) for finger bending monitoring, a T‐TENG sensor (Figure 1a‐iii) for contact position and area detecting, and a PVDF sensor (Figure 1a‐iv) for temperature sensing. The T‐TENG sensor made of silicone rubber shows good flexibility, which can fit and follow the pneumatic fingers well under different deformation conditions. The L‐TENG sensor measures the bending angle by counting output peaks, capable of avoiding environmental influences (humidity, temperature, etc.). The PVDF sensor detects the temperature of grasped objects based on pyroelectric output amplitude with high linearity in a wide temperature range, and the temperature resolution can be as low as 1 °C. The integrated multifunctional sensory system mimics the biological sensory system of human skin (Figure 1b), which contains various receptors responding to different physical information, i.e., L‐TENG‐based strain sensing, T‐TENG‐based tactile sensing, and PVDF‐based temperature sensing. By integrating the tactile and strain sensory information from the sensors at the data level through the IoT module, ML enhanced data analytic can be leveraged to help the manipulator realize more complex perception functions. As a result, the recognition of grasped objects is realized as a validation feedback in the digital‐twin‐based virtual shop application. Furthermore, the temperature sensory data will be incorporated at the decision level, more comprehensive information of the grasped objects can be obtained to provide the actual status of the item in real space. Compared with the current flexible solutions listed in Table S1 (Supporting Information), the proposed fully self‐powered solution shows its unique advantages considering the diversified sensing functions, i.e., tactile, deformation, temperature sensing, that have been achieved in the one integrated system. With the further exploration of the proposed digital‐twin‐based sensory interactive system, there will be a promising potential of enhancing intelligence and efficiency in various applications, ranging from healthcare to industrial automation.

## 2 Design and Working Mechanisms of the Soft Manipulator Using Self‐Powered Sensors

### 2.1 Working Mechanism of the Pneumatic Actuator

The soft‐robotic manipulator consists of three pneumatic actuators as shown in Figure 1a‐i, and the working mechanism and the detailed hollow‐bellows design of the actuator are depicted in Figure S1a,b (Supporting Information). The top of the pneumatic actuator is a corrugated structure and the bottom is a flat surface. The whole soft actuator is directly printed by the 3D printer, and the technical parameters of the 3D printing process can be found in Table S2 (Supporting Information) and Experimental Section. As the device is inflated by air, the top of the actuator will have a larger deformation due to the relatively lower stiffness compared to the bottom, resulting in the bending of the soft actuator, as shown in Figure S1c (Supporting Information). Besides, when the cavity is inflated, it will bulge, causing the adjacent sidewalls to contact and increase the deformation of the actuator. The bending angle of the actuator is nearly linear with the applied air pressure within a specific range, which has been proven by the previous works^[^ ^114^ ^]^ and can be used to precisely control the degree of deformation of the soft actuator. When a certain amount of air pressure is applied, the three actuators of the soft manipulator will bend simultaneously, thereby exerting a three‐sided balanced contact force on the object and realizing the function of grasping.

### 2.2 Working Mechanisms of the TENG Sensors

**Figure 2:**
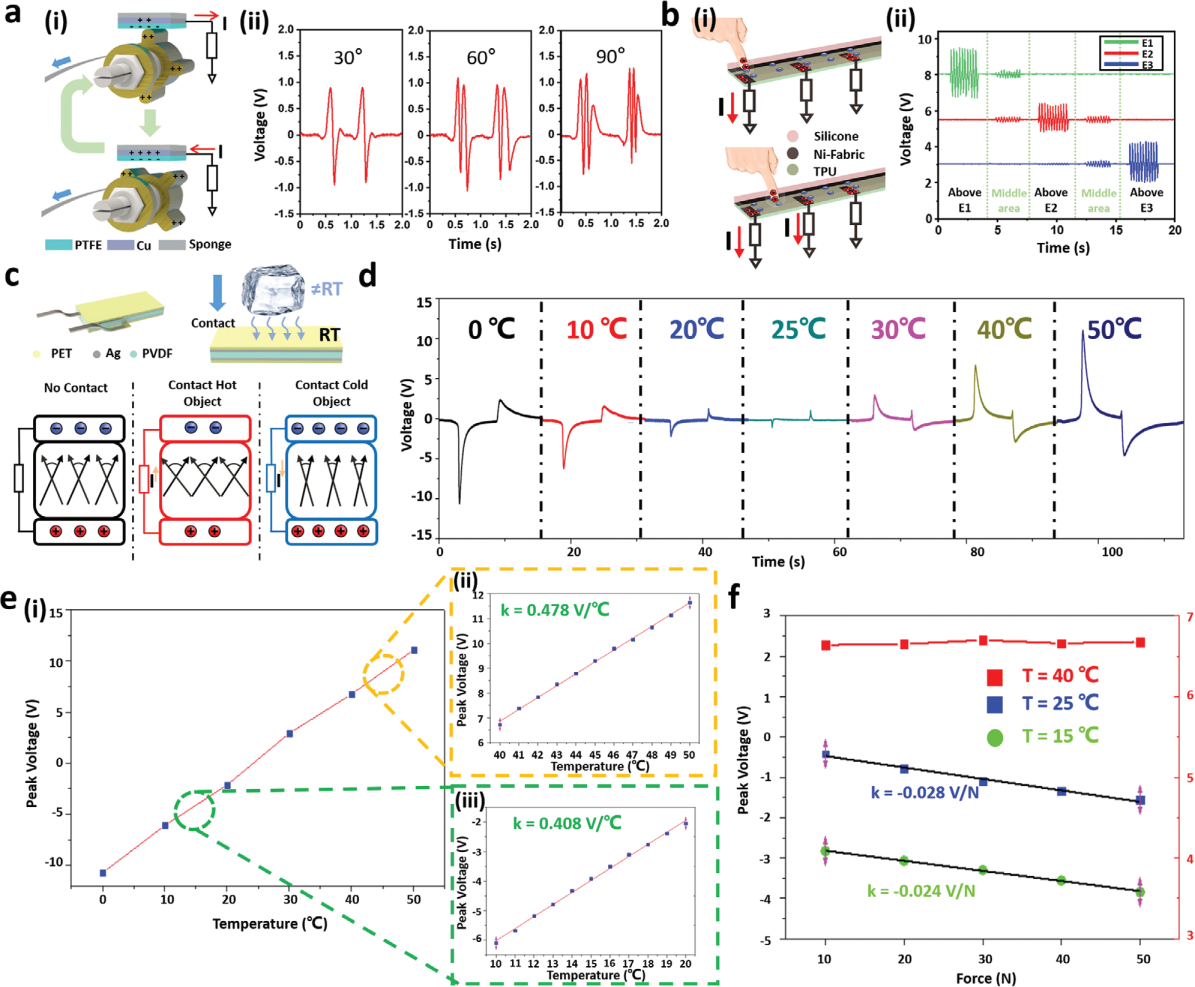
Working mechanisms of integrated sensors. The working mechanism and the corresponding output signal of a) L‐TENG sensor and b) T‐TENG sensor. c) The working mechanism of pyroelectric‐based PVDF temperature sensor and d) its piezoelectric‐pyroelectric superimposed output when contacting with TEC under different temperatures with contact force of 40N. e‐i) The calculated peak values of the PVDF sensor during contact motion when the temperature of TEC equals to 0 °C, 10 °C, 20 °C, 30 °C, 40 °C, and 50 °C. The temperature sensing performance test of the PVDF sensor in the temperature range of e‐ii) 40 °C – 50 °C and e‐iii) 10 °C–20 °C with 1 °C intervals. f) The influence of the pressure‐induced output on temperature sensing.

The structure of the L‐TENG sensor is shown in Figures 1a‐ii and **2**a‐i. A gear is mounted on the metal shaft, with a layer of Ni‐fabric conductive textile covered on the gear surface. One end of the strip is fixed on the shaft, and the other end is fixed on the fingertip of the pneumatic actuator. When the pneumatic actuator is inflated and deforms, the strip will be stretched and drives the gear to rotate, resulting in intermittent contact between the Ni‐fabric covered gear teeth and PTFE thin film. Due to triboelectrification on the contact surface and electron affinity difference, the PTFE thin film tends to attract electrons and hold them on the surface, while the gear teeth tend to lose electrons and become positively charged. During the following rotation and contact‐separation process, the variation of the electrical potential from the electrostatic induction between the electrodes and the ground will drive electrons flow and generating the triboelectric output peaks as shown in Figure 2a‐ii. Here, one output peak means a complete cycle of contact and separation between a single tooth of the gear and the PTFE film, and a bending angle of 30° of the pneumatic actuator. So the continuous deformation of the finger can be measured according to the output peak numbers, and the resolution of the L‐TENG sensor could be further increased with increasing gear size and gear teeth number. Besides, the disc spring connected with the shaft is compressed when the pneumatic finger is inflated and will be released and provide the pull‐back force for the pneumatic finger to recover when air goes out, making the manipulator respond more quickly and ready for the next gripping.

For the design of the T‐TENG sensor (Figures 1a‐iii and 2b‐i, four distributed electrodes are attached to the TPU substrate, with a layer of silicone rubber covered on the top. Three short electrodes (5 mm × 20 mm, labeled as E_1_‐E_3_) are arranged at intervals of 20 mm for contact position detecting, and the long electrode (5 mm × 100 mm, labeled as E_L_) is attached along the long edge of the T‐TENG patch for contact area measuring. When the silicone rubber contacts with other objects, charges will be induced in these four distributed electrodes due to triboelectrification and electrostatic induction. The output voltage amplitude in each short electrode is inversely proportional to the distances between the electrodes and the contact point. As plotted in Figure 2b‐ii, when the contact position is right above E_1_, the most charges will be induced in E_1,_ and the output amplitude is the highest among the three short electrodes. However, when the contact position is between two adjacent short electrodes (E_1_ and E_2_), both electrodes will have outputs. Based on this feature, the contact position on the T‐TENG patch can be determined by further calculating the voltage ratio of the three short electrodes, as depicted in Figure S2a (Supporting Information). When the ratio of one short electrode output to the total output of the three short electrodes ($\mathrm{Ratio}=V_{i}/\sum_{i=1}^{3}V_{i}$, i means *i* ^th^ electrode) is higher than 0.8, the contact position can be regarded as being directly above the electrode. When the ratio value is between 0.3 and 0.7, the contact position can be seen as being between two adjacent electrodes. The resolution of the position detecting is 10 mm considering the 20 mm interval between two short electrodes and can be further increased by decreasing the interval distance and attaching more short electrodes along the length direction of the patch. In addition, different contact areas will also induce different amounts of charges in E_L_. As shown in Figure S2b (Supporting Information), when the external stimuli occur at different locations on the patch surface in the form of point contact with the same contact force, there is almost no difference in the output amplitudes of E_L_ since the approximate contact area. However, the increase of the contact area will generate more charges in E_L_. The output voltage amplitude is nearly proportional to the contact area (Figure S2c, Supporting Information), which can clearly reflect the variation of contact area during grasping and used to perceive the gripping mode of the soft manipulator, i.e., point contact or area contact.

### 2.3 Working Mechanism of the PVDF Temperature Sensor

Temperature sensing is another important function by considering the relative higher or lower temperatures of many items against the room temperature, e.g., frozen meat, iced drink, and hot coffee, etc. This information is valuable for customers to have a thorough understanding of the good and choose the product that best fits their preference. In order to be compatible with the nonlinear deformation of soft robots, the polyvinylidene fluoride (PVDF) sensor with high flexibility is chosen and integrated with the smart manipulator serving as the temperature sensing unit based on the pyroelectric mechanism. Figures 1a‐iv and 2c show the detailed structure of the PVDF sensor, including a poled PVDF film with Ag electrodes on both surfaces and packaged with polyethylene terephthalate (PET) thin film. Similar to the T‐TENG sensor panel, the PVDF sensing unit is also attached to the inner surface of the pneumatic finger and utilized for generating the temperature‐related output when in contact with the external objects. The strain on the PVDF film will also induce pressure‐related output due to the piezoelectric property of the PVDF material during the collision process. Decoupling the pressure influence for obtaining the accurate temperature output is then required to be solved. The working mechanisms of piezoelectric and pyroelectric can be found in Figure S3 (Supporting Information) and Figure 2c, respectively. As depicted in Figure S3 (Supporting Information), the piezoelectric output can be generated by the polarized PVDF sensor under the compressive/tensile stress due to the change of the polarization intensity. However, for the mechanism of pyroelectric shown in Figure 2c, the output generated based on the thermally induced random swing of the electric dipole around its balance axis. When the polarized PVDF film cools, since the electric dipole oscillates within a smaller spread angle due to lower thermal activity, the spontaneous polarization will be enhanced. Then due to electrostatic induction, the variation of the spontaneous polarization can drive electrons to flow between the two electrodes and generate the output. When the temperature applied to the polarized PVDF film rises, the spread of the electric dipoles on their respective alignment axes becomes greater, reducing the spontaneous polarization and resulting in the electron flow in the opposite direction.

To test the temperature sensing performance of the PVDF film, a force gauge and a thermoelectric cooler (TEC) are utilized to simulate the situation of contact with objects under different temperatures, and the detailed set up can be found in Figure S4 (Supporting Information), where the PVDF sensing unit (12 mm × 22 mm) is attached to the moving load cell of the force gauge and contact with a fixed TEC whose temperature can be controlled by the TEC controller. Figure 2d shows the output voltage of the PVDF film when in contact with the TEC under different temperatures with the same contact force of 40 N. When there is no temperature difference between the TEC and PVDF sensing unit (TEC temperature = 25 °C, room temperature), the output is purely generated by the contact pressure, where the contact motion induces a negative peak, and followed by a positive peak when the PVDF film and TEC are separated. When the temperature of the TEC cools down, and lower than that of the PVDF film (TEC temperature = 20 °C, 10 °C, 0 °C), a larger and wider negative peak is generated due to the temperature difference based on pyroelectric when contact happens, whose direction is the same as that of the output caused by pressure, and shows a superimposed effect between the piezoelectric and pyroelectric outputs. Besides, it can be seen that the negative peak value increases as the temperature difference increases. However, when the temperature of the TEC rises and higher than the room temperature (TEC temperature = 30 °C, 40 °C, 50 °C), a positive voltage peak is generated during the collision due to the greater spread of the electric dipoles on their respective alignment axes in PVDF, whose peak direction is opposite to that of the pressure, as well as the output caused by the lower temperature, and completely counteract the piezoelectric output as shown in the right half of Figure 2d. Figure 2e‐i depicts that the pyroelectric peak value is approximately linear in the temperature range of 0 °C to 50 °C and increases with the increasing temperature. Figure 2e‐ii,iii demonstrate the linearity and sensitivity of the PVDF sensing unit under high temperature and low‐temperature sensing, respectively. Even measured at intervals of 1 °C, the variation of the temperature can still be clearly distinguished, showing the strong temperature perceiving ability of the PVDF sensor.

To further investigate the influence of the contact pressure on temperature sensing, the PVDF film is tested with different contact forces under room temperature first, and the generated outputs are plotted in the black line with blue squares in Figure 2f. The result shows that the negative piezoelectric output amplitude is linear with the contact force and increases from 0.42 to 1.56 V with a slope of 0.028 V N^‐1^ as the contact pressure increases from 10 to 50 N. Due to the superimposed effect mentioned above, the varying piezoelectric outputs under different contact pressures may affect the pyroelectric–piezoelectric superimposed outputs, resulting in the error of the temperature perception in terms of low‐temperature objects. As the black line with green dots in Figure 2f shows, when the TEC temperature is maintained at 15 °C, the output peak values change with the contact pressure. The trend and degree of the variation are similar to that of the outputs purely induced by pressure, proving the influence of contact pressure changes on low‐temperature measurement. However, when the temperature of TEC is higher than the room temperature and maintained at 40 °C as depicted in the red line in Figure 2f, there is no obvious change in the output amplitudes under different pressures, indicating that the contact pressure nearly has no influence on the high‐temperature sensing. The different effects of the contact pressure on the high/low‐temperature sensing may result from the different response time of the pyroelectric output at high/low temperatures, as shown in Figure S5 (Supporting Information). It is clear that the response time in the high‐temperature zone (temperature higher than the room temperature) is generally longer than that of the low‐temperature zone (temperature lower than the room temperature). The shorter response time under a low‐temperature situation gives rise to more pressure output superimposed on the pyroelectric peak, thus resulting in a greater impact. Combining the results from Figure 2f,e‐iii, an error of 0.06 °C N^‐1^ can be calculated for a low‐temperature sensing situation (*T* = 15 °C), which is acceptable considering the small variation of the contact pressure when grasping objects by the soft manipulator. Besides, in Figure S5 (Supporting Information), we can also find that when the temperature continues to decrease (*T* = 10 °C, 0 °C), the response time will increase slightly, so that the influence and error induced by the pressure will also be reduced accordingly.

## 3 ML‐Enabled Automatic Grasped Objects Recognition System

**Figure 3:**
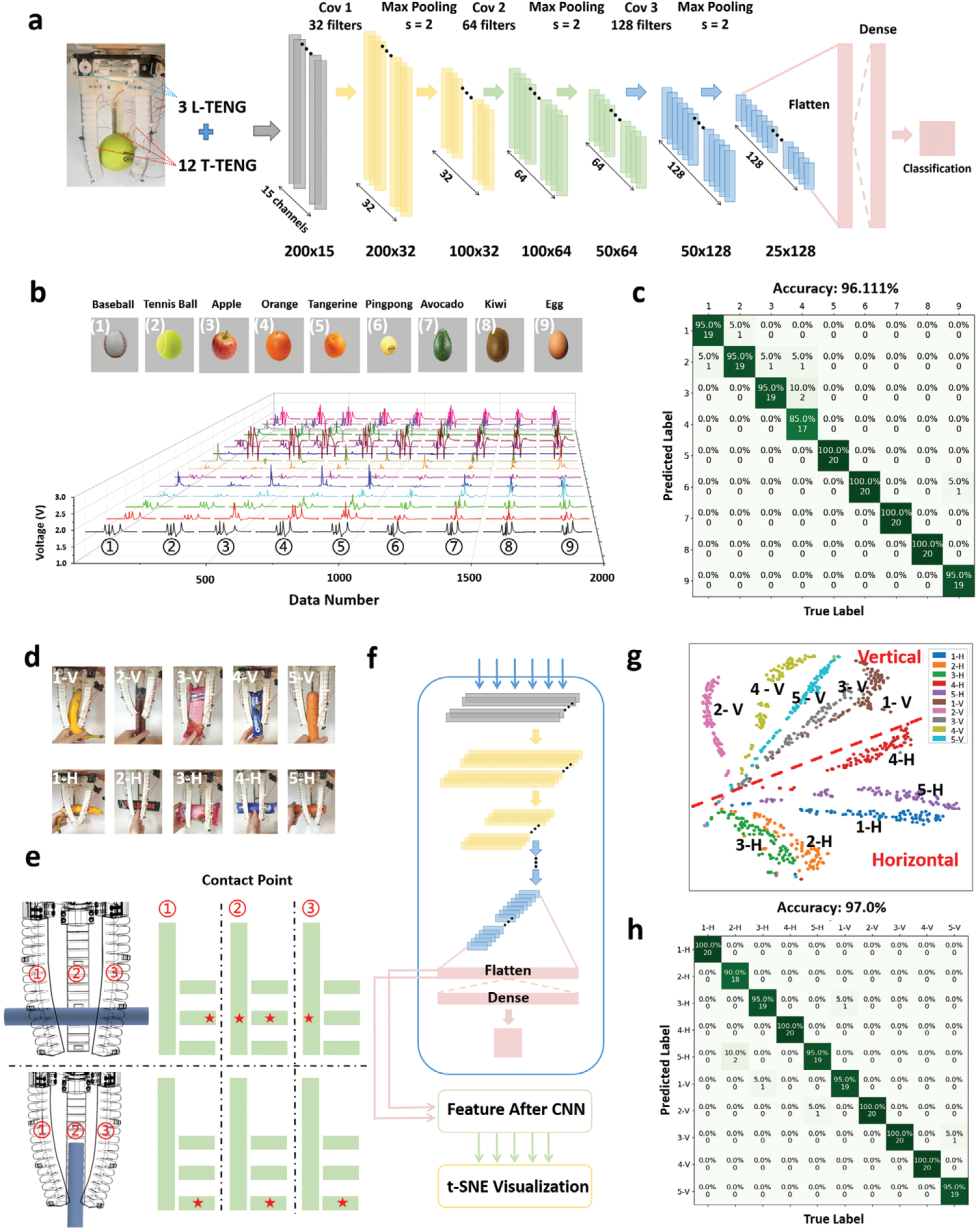
ML‐enabled automatic grasped objects recognition system. a) The detailed framework of the 1D‐CNN ML analytic. b) The typical 15‐channel output spectra collected by the integrated TENG sensing system of 6 spherical and 3 oval objects as the input of the ML algorithm. Columns of 15‐channel spectrums labeled as 1–9 from left to right corresponding to the signal of grasped objects labeled as 1–9. c) The corresponding confusion map for the 6 spherical and 3 oval objects after recognition. d) The diagrams of the manipulator grasping 5 elongated objects in vertical and horizontal manner. e) The deformation condition and contact point map of the manipulator for grasping elongated object horizontally and vertically. The marks of five‐pointed star represent the contact positions on the T‐TENG sensor patches integrated on three pneumatic fingers f) The detailed framework of the t‐SNE visualization process. g) The t‐SNE visualization results of the data from both grasping vertically and horizontally. 1V–5V and 1H‐5H represent the data points of the 5 elongated objects grasped vertically and horizontally respectively. h) The confusion map of the 5 elongated objects under two different grabbing angles (grasping vertically: 1V–5V and grasping horizontally: 1H–5H).

The above results prove that the L‐TENG and the T‐TENG sensors are applicable to be used for contact area, position, and bending angle detection for soft manipulators due to their high flexibility and good compatibility with soft robots' no‐joint deformation. By integrating three L‐TENG sensors and three T‐TENG sensors with a tri‐finger pneumatic gripper, a smart soft robotic manipulator is developed, as illustrated in Figures 1a‐i and **3**a. Each L‐TENG sensor has one channel, and each T‐TENG sensor has four channels. There are 15 channels in total for grasping data collection. When the objects are grasped by the soft manipulator, the stimuli applied on the inner surface of each finger will generate a three‐dimensional sensory information that contains the contact positions and areas of the three contact surfaces. The deformation of each finger also contains information about the sizes and shapes of the grasped objects, especially for the asymmetric objects. Unlike the individual values of contact position, contact area, and bending angle calculated from the sensing signal, the original 15‐channel spectrum in the time domain of one gripping motion may contain more hidden information, including contact sequence, collision vibration, bending speed, etc. This information is a valuable feature of each sample for better classification. Moreover, machine learning methods, including support vector machine (SVM),^[^ ^115^ ^]^ one‐dimensional convolutional neural network (1D‐CNN),^[^ ^7^, ^92^, ^108^ ^]^ deep belief network (DBN),^[^ ^116^ ^]^ etc., have been proven as effective tools that can automatically extract the features from the time domain data of the triboelectric signals and make the identification between different samples with high accuracy. As shown in Figure 3a, a three‐layer 1D‐CNN was then constructed for data feature extraction and automatic recognition to verify the sensing ability of the proposed intelligent manipulator system. The detailed parameters of the ML architecture can be found in Table S3 (Supporting Information).

A data set contains six spherical objects, including baseball, tennis ball, apple, orange, tangerine, and ping‐pong, and three oval objects, including avocado, kiwi, and egg, with similar shapes, is established by repeating grasping of each object by 100 times, as illustrated in Figure 3b. The data length for each channel is 200, so there are 15 channels * 200 = 3000 features in total for each sample as the input of the 1D‐CNN analytic. The 100 samples of each object are randomly divided into training and testing set at a ratio of 8:2. Though the output voltage spectra of the six spherical objects and three oval objects look similar and difficult to be directly classified through the output signal as plotted in Figure 3b. After 50 epochs of training, the testing accuracy of these nine objects can reach up to 96.111%, showing the powerful perceiving ability of the proposed learning architecture and sensing system when encountering objects with approximate shapes. In the confusion map (Figure 3c), it can be found that the most errors occur between baseball, tennis ball, apple, and orange, which is acceptable considering the similar sizes and shapes of these four spherical objects.

Different from grasping spherical objects, for which the grabbing angle nearly has no effect on the output signal, different grasping angles of the same elongated object will result in different contact positions on the T‐TENG sensor panels (Figure 3e), as well as different deformation of the three pneumatic fingers, thus generating various output spectra for the same elongated object. In addition, when different elongated objects are grasped with the same angle, the similar contact position, contact area, and fingers’ bending angle caused by their approximate diameter also increases the difficulty of recognition. Here, five elongated objects are selected to be grasped vertically and horizontally as illustrated in Figure 3d. Though their shapes and sizes have visible differences, it is difficult to directly distinguish these five elongated objects from the output spectrum due to the similar contact position under the same grasping angle as plotted in Figure S6a,c (Supporting Information). However, after the feature extraction process of the construed 1D‐CNN learning architecture, the t‐distributed stochastic neighbor embedding (t‐SNE) algorithm as a nonlinear dimensionality reduction technique well‐suited for embedding high‐dimensional data for visualization in a low‐dimensional space of two or three dimensions is successfully utilized.^[^ ^117^ ^]^ It can reduce the dimensionality of extracted features achieved by 1D‐CNN (Figure 3f) and visualize the clustered results of the data set of vertical and horizontal grip, respectively, as shown in Figure S6b,d (Supporting Information). In Figure S6b (Supporting Information), it is clear that the 100 sample points of each object grasped vertically are clustered together, proving that the proposed smart manipulator system is able to sense the subtle differences hidden in the waveform between different categories and make the correct perception. By connecting a layer of softmax for further identification, the testing accuracy of the vertical grip can reach 98.0% with only two errors in 100 testing samples, as depicted in Figure S7a (Supporting Information). Similarly, a high perceiving accuracy of 97.0% can also be achieved for the horizontal grip as shown in the confusion map in Figure S7b (Supporting Information).

By combining these two data sets of different gripping angles into a whole data set, the clustered result of the 10‐category data set is visualized in Figure 3g. Though the data points from the same object under the same gripping angle are clearly gathered together, the data of the same object's two gripping angles is divided into two clusters, showing that the learning architecture has identified the different angles of the same object into different categories due to the varying contact positions, contact areas, and pneumatic fingers’ deformation degrees. A clear dividing line can be seen in Figure 3g between the five horizontally gripping and five vertically gripping data points, indicating that the generalized gripping angle for elongated objects can also be detected by the smart sensing system. When an unknown elongated object is grasped, this object could be classified into a big category according to the grasping angle first, then processed in the category without the influence of the gripping angle for better classification. The confusion map in Figure 3h demonstrates the high recognition accuracy of 97% for the combined data set by the constructed learning architecture. Most errors occur within the same gripping angle, which is reasonable considering the similar contact and bending situation. Moreover, if we fuse the data of the same object under different grasped angles into the same class and tag with one label, then retraining, the perceiving accuracy is still as high as 96.0% as shown in Figure S7c (Supporting Information), meaning that the effect of gripping angles could be avoided by collecting more data of different grasped angles for the same elongated object to make the data set more generalized.

## 4 Online Virtual Shop Application

**Figure 4:**
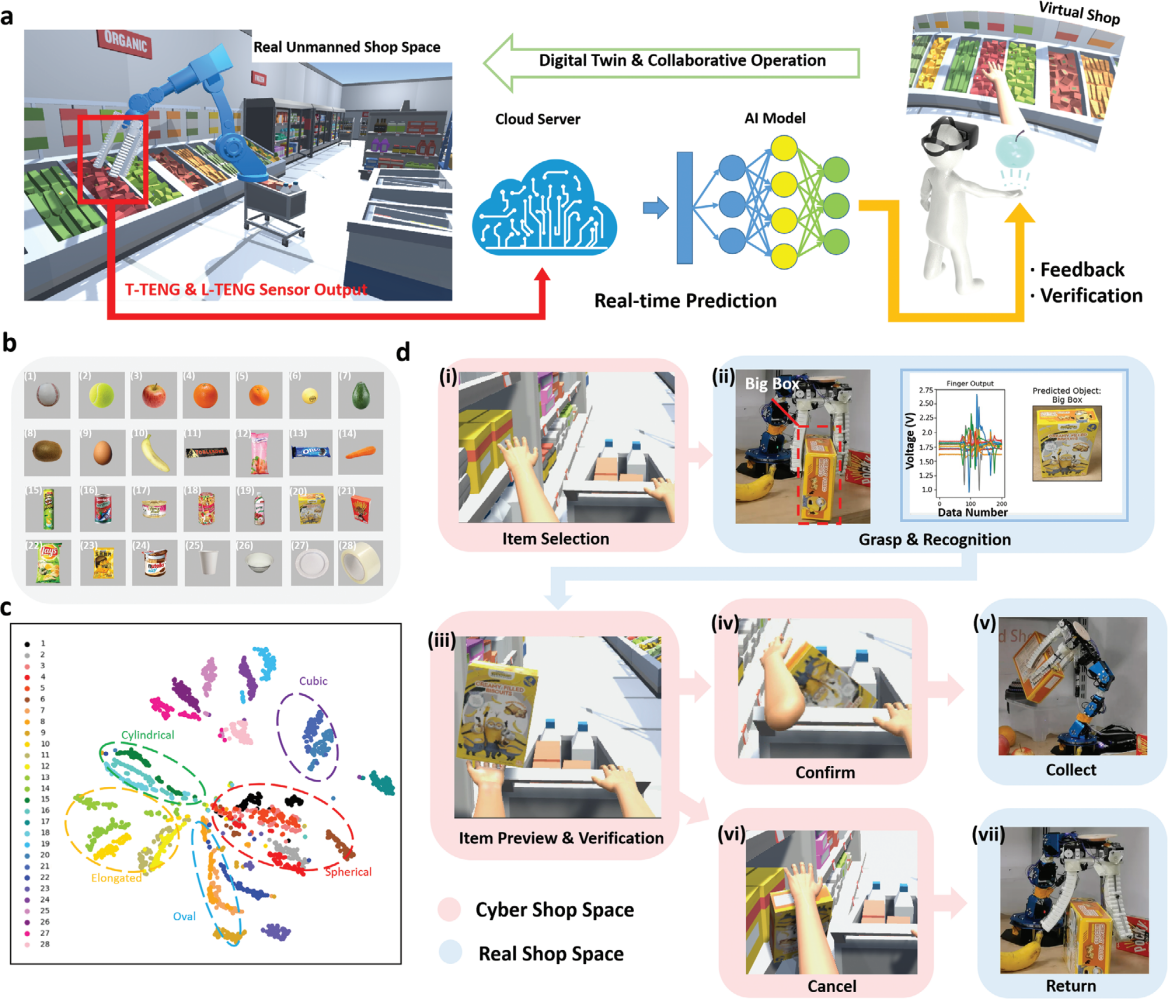
The demonstration of the digital‐twin‐based virtual shop application. a) System architecture of the digital‐twin‐based virtual shop. b) The 28 objects with different shapes and sizes to be identified by the system. c) The t‐SNE visualization of the clustered result of the data set including 28 categories. d) The real‐time system interface with the testing object of a big box. The processes with red background represent the user operations in the cyber space. The processes with blue background represent the robot motions in the real unmanned space.

With the rapid development of the Internet and logistics, online shopping has become an indispensable part of our daily lives, which brings us great convenience and helps us collect favorite products without going out. Besides, with the gradual rollout of AIoT technology, AI robots have the potential to replace humans and become the main force in the simple working environments, e.g., assembly factories, shops, etc. To show the potential of the proposed intelligent manipulator for future online shopping and unmanned shop applications, we propose a digital‐twin‐based virtual shop system, as illustrated in **Figure** **4**a. With this tool, users can have an immersive shopping experience in the digital‐twin virtual store enabled by VR technology, which duplicates and simulate the real space of the physical store. In the meantime, an intelligent robotic manipulator in the real unmanned shop space will perform coordinated movements corresponding to the motions and selections from the user side, then grasping the chosen good and making the perception according to the sensory information of sizes and shapes collected from the TENG sensors. The predicted results from the manipulator side can be real‐time transmitted back to the user side and utilized to reconstruct the selected good in VR space for feedback and validation purposes.

In order to make our system applicable to the normal store situation, we enlarged the data set with more common goods in various shapes, including cubic, elongated, cylindrical, oval, spherical, etc., as illustrated in Figure 4b. The number of categories is increased to 28, which is much larger than the data set composed of 16 objects reported previously,^[^ ^114^ ^]^ and the corresponding t‐SNE visualized result is plotted in Figure 4c. It is obvious that the data points from each good are clustered together, with some overlaps between several categories. This overlapping phenomenon does not mean that these data cannot be distinguished by the machine learning algorithm. The result of this aggregation is obtained through two‐dimensional features for visualization, and it is difficult for some similar categories to be identified just based on only two‐dimensional features. When using more extracted features for classification, the high recognition accuracy of 97.14% can still be achieved as shown in Figure S8 (Supporting Information), even though the number of categories is as high as 28, showing the applicability of the ML‐enabled smart manipulator to various object shapes. In Figure 4c, except for the aggregation of the data from each object, the data points from similar shapes, i.e., elongated, cubic, cylindrical, oval, and spherical, are also gathered together and circled with different colors. This phenomenon indicates that when the data set is more generalized with massive injected categories and data, unknown objects for the trained model could still be identified based on their specific shapes: cubic object, spherical object, oval object, etc., demonstrating the applicability of the sensing system in more general object recognition. The stability test of the integrated sensory system can be found in Table S4 (Supporting Information). Though there are some fluctuations in the accuracy when the utilization cycles reach 500, 1000, 1500, and 2000, there is no obvious decline in the accuracy after continuous testing for two thousand times, proving the stability of our proposed sensory system for long‐term use.

The specific operation details of the virtual shop system can be found in Figure 4d and Movie S1 (Supporting Information). In Figure 4d‐i, a big box that existed in the data set is selected and grasped by the user in the virtual space first. Then the soft manipulator in the real unmanned shop space will grasp the same product according to the user's movement and selection, generating a 15‐channel output spectrum collected from the T‐TENG and L‐TENG sensor as plotted in Figure 4d‐ii based on the actual contact and deformation conditions of the pneumatic fingers. Next, the trained ML‐enabled analytic model based on the data set of 28 objects makes the identification according to the 15‐channel output spectrums, and real‐time transmit the feedback information and reconstruct the virtual objects with the same size and shape in VR store space. The generated virtual goods are floating above the user's hand in the VR space as shown in Figure 4d‐iii, where the user can turn his hand and have a more detailed observation of the goods from all directions. This function offers a more intuitive impression of the products, as well as the feeling of choosing goods on‐site. Besides, the user will also be able to know whether the robotic manipulator grasps the correct product or not as a validation function considering the possible errors between the real and virtual spaces caused by the delay of the status update in real space. After verification, the user can easily make the final decision by putting the object directly to the trolley or back to the shelf in the virtual space as illustrated in Figure 4d‐iv,vi, triggering the collaborative operations of the robotic arm in the real space to move the chosen product to the physical trolley or just leave at its original position (Figure 4d‐v,vii). The operation details are demonstrated in Movie S1 (Supporting Information). Such an interactive feedback system can help users to interact with the real space remotely in real‐time while giving them an immersive operating experience.

## 5 Virtual Shop User Interface Enriched by Temperature Sensing Information

**Figure 5:**
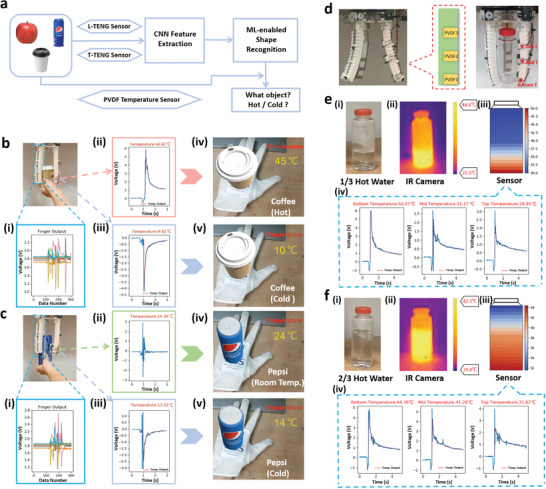
Unmanned shop temperature sensing. a) The flow chart for the shape‐size‐temperature fused sensory system. The real‐time system interface with testing object of b) hot/cold coffee and c) room temperature/iced canned drink. d) Illustration of the manipulator integrated with three PVDF sensors for temperature distribution sensing. The temperature distribution map and the corresponding sensor outputs achieved by the IR camera and PVDF sensors for e) 1/3 bottle of hot water and f) 2/3 bottle of hot water.

Apart from the tactile and deformation perception function, the ability of temperature sensing is also an important part of realizing the anthropomorphic robotic finger that simulates the multifunctional human biological perception system. Thus, we integrate the flexible PVDF temperature sensor onto the smart manipulator, as illustrated in Figure S9 (Supporting Information). A piece of sponge is attached between the PVDF sensing film and the pneumatic finger to increase the contact area on the PVDF sensor surface during grasping, considering the bulged surface of the inflated finger. In order to reduce the mutual influence between the PVDF and T‐TENG sensor, one pneumatic finger is integrated with the PVDF sensor for temperature detecting, with the remaining two fingers integrated with both of the TENG sensors for shape and size perceiving. Though the channel number of the TENG sensors has been reduced from 15 to 10, and the shape/size related information of one face of the grasped object is lost, the confusion map in Figure S10a (Supporting Information) shows that a high identification accuracy of 100% can still be achieved for apple, drink can, and cup illustrated in **Figure** **5**a based on a new 10‐channel data set. In addition, if a 10‐channel data set is extracted from the original 15‐channel data set of the 28 objects for retraining, the accuracy of 95.89% can still be maintained (Figure S10b, Supporting Information), proving the effectiveness of the 10‐channel information for object shape perception. To verify the influences of finger numbers on the recognition accuracy, we have done the additional test as shown in Figure S11 (Supporting Information), where only one finger is integrated with the TENG sensors and the accuracy is 92.857% for the five‐channel data set. To conclude, when the number of TENG sensor‐equipped fingers increased from one to two, the accuracy rate of 28 objects has increased by more than three percentage points. However, when the number of fingers equipped with the TENG sensors increased from two to three, the accuracy increases only about one percentage point at the expense of much more data from five channels and 1.5 times the data set size. If we continue to increase the number of fingers equipped with sensors, the accuracy rate will definitely increase, but the profit will be relatively lower, considering the increase in the data set size as well as the cost in data processing.

Taking into account that the temperature distribution of many goods themselves is relatively uniform, a piece of PVDF sensor (12 mm × 22 mm) is first attached to the fingertip of the pneumatic finger to detect the overall temperature of the products, and the architecture of the TENG–PVDF integrated system is shown in Figure 5a. When the object is grasped by the smart manipulator, the ML‐enabled analytic will perceive the shape/size of the object first according to the 10‐channel signal spectrum collected by TENG sensors. After this step, the grasped object can be successfully classified into different categories based on its shape/size. Then the output signal of the PVDF sensor can be utilized to further fuse the temperature‐related information with the identification result at a decision level. It is worth mentioning that, when the PVDF sensor is mounted on the soft manipulator, the collision speed during grasping is faster than that of using the force gauge, resulting in a much faster generation of the piezoelectric output than the pyroelectric output, and less superimposed effect as plotted in Figure S12c,d (Supporting Information). Besides, due to the electron affinity difference between the PVDF packaging material (PET) and various objects, the triboelectric output will also be generated during the collision. However, such collision‐induced output peaks are always generated before the temperature output and can be easily separated as Figure S12c,d (Supporting Information) shows, so the interference to the pyroelectric output peak caused by the collision could be ignored in this case. Figure 5b,c demonstrates the real‐time shape/size/temperature perception of the smart system for unmanned shop applications, where cups of coffee and canned drinks with two different temperatures are tested. The soft manipulator grasps the cup or the canned drink according to the user's selection first. Then based on the 10‐channel sensory spectrum plotted in Figure 5b‐i,c‐i, the grasped object is successfully recognized as the cup of coffee or canned drink according to its shape/size. Next, with the temperature information calculated from the pyroelectric output amplitude shown in Figure 5b‐ii,iii,c‐ii,iii, the grasped objects can be further classified into cold/hot coffee or room temperature/iced canned drink as illustrated in Figure 5b‐iv,v,c‐iv,v, respectively. The pyroelectric outputs here are collected by the IoT module, whose values are slightly different from those directly collected by the oscilloscope mentioned in the sensor characterization part, and needed to be recalibrated as Figure S12a,b (Supporting Information) shows. The operation details can also be found in Movie S2 (Supporting Information). Besides, the result in Table S5 (Supporting Information) shows that the object temperature nearly has no influence on the TENG sensor‐based object recognition, where the coffee cup and the Pepsi can are repeatedly grasped 20 times under different temperatures to test the classification accuracy.

Expert for objects with uniform temperature distribution, some objects, e.g., 1/2 cup hot/cold water, etc., have temperature variation in different parts of the body. In view of this situation, distributed temperature sensor nodes are needed to be arranged onto the manipulator to capture the temperature distribution across the object and reflect its accurate temperature information. Here, we integrate one pneumatic finger with three PVDF temperature sensors as illustrated in Figure 5d to detect the temperature of three contact points during grasping: bottom, mid, and top. By dividing the temperature gradient between two adjacent detecting points equally, a rough temperature distribution map could be achieved as shown in Figure 5e,f, and Movie S3 (Supporting Information). In Figure 5e, 1/3 bottle of hot water is grasped and perceived by the smart manipulator, and the real‐time temperature distribution map of the bottle is captured by the infrared (IR) camera shown in Figure 5e‐ii. According to the pyroelectric outputs generated in the three distributed PVDF temperature sensors during grasping as plotted in Figure 5e‐iv,iii illustrates another temperature map that calculated based on the collected sensory information, which shows high similarity to the results achieved by the IR camera, verifying the temperature distribution sensing function of the proposed system. Similarly, when the hot water in the bottle increased to two thirds (Figure 5f‐i), the hot zone area in the temperature distribution graphs obtained by the IR camera and PVDF sensor increases simultaneously and remains consistent, as shown in Figure 5f‐ii,iii, proving the reliability of the temperature distribution information achieved by the PVDF sensors.

In addition to the shape and temperature, there are also other aspects that may be important for shopping, such as the freshness, which still requires further research to enrich the functionalities of the sensory system. For instance, as a solution to identifying the freshness, the gas and chemical sensing technology are utilized.^[^ ^118^ ^]^ The corresponding wearable solutions with high flexibility for food security and industrial applications have attracted great attention in recent years,^[^ ^119^ ^]^ showing the great compatibility to be integrated with the current embedded sensory system to realize the freshness detection function for the soft manipulator without the help of the camera. On the other hand, the application of the camera to capture the shape, temperature, and freshness‐related information, i.e., color and sign of defects, is definitely a straightforward solution with the tradeoff of a large volume of data which required greater transmission bandwidth and computing capacity. The embedded sensory system still could be a complementary solution for the visual images by providing more shape and temperature‐related information from other dimensions (non‐visual) to enhance the performance of the intelligent system after adopting the deep learning enabled data fusion technology.

## 6 Conclusion

In general, a soft robotic manipulator integrated with an L‐TENG sensor for bending angle detecting, a T‐TENG sensor for contact position and area sensing, and a PVDF sensor for temperature sensing is developed, which can mimic the complex and multifunctional biological perception system of human skin and realize automatic object recognition function. By integrating three sensors on one pneumatic finger, temperature distribution information of the grasped object can also be achieved, which enriches the perception function of the robotic finger and gives users a more comprehensive understanding of the product. With the help of the 1D‐CNN ML algorithm for automatic feature extraction, the classification accuracy for 6 spherical and 3 oval objects can reach up to 96.1%, showing the identification performance of the proposed system for objects with similar shapes and sizes. By tagging the same object grabbed from different angles with the same label, the influence of grasping angle can be avoided. This integrated system is also applicable to other shapes of objects, e.g., cubic, cylindrical, etc., and high identification accuracy of 97.143% is achieved for 28 different shapes of goods. With the improved intelligence of the soft manipulator, a virtual shop which is able to real‐time synchronize user's intended actions in the VR space and robotic arm's motion in the real space with AIoT platform, as well as providing accurate feedback information of products with robot's integrated multifunctional system, is successfully implemented to provide more immersive online shopping experience. Moreover, with the fusion of the temperature sensory information from the PVDF sensor and the TENG tactile sensory data at the decision level, the further interpretation of the objects related to the temperature distribution can be achieved in addition to the shape recognition. As a future prospect under the 5G and AIoT infrastructure, by utilizing the self‐powered sensory interactive system with the features of the facile design, low cost, and high compatibility, etc., together with ML techniques, a smart society can be established through the intelligent industrial automation, shopping, education, healthcare, etc.

## 7 Experimental Section

### Fabrication of the TENG‐Based Sensors

The short and long electrodes of the T‐TENG sensor were made of Nickel conductive textile, and the dimensions were 5 mm × 20 mm and 5 mm × 120 mm, respectively. These electrodes were attached to the surface of the TPU substrate first, then poured a layer of mixed solution on top with equal amounts of A part and B part of the EcoFlex 00‐30. After baking at 50 °C for 20 min, the mixed solution will form into a layer of flexible film and become the negative triboelectric layer of the T‐TENG sensor. The area of the T‐TENG sensor patch is 26 mm × 120 mm. The L‐TENG sensor's main structure was directly printed by a 3D printer (4max pro, Anycubic), with a layer of Nickel conductive textile covered on gear teeth and a layer of embedded PTFE layer as the positive and negative triboelectric material respectively.

### Fabrication of the Soft Pneumatic Actuator

The bellows‐structured pneumatic actuator was designed with a solid modeling software: Solidworks 2016, and fabricated by 3D printing with a commercialized 3D printer (4max pro, Anycubic) using TPU filament (NinjiaFlex, hardness of shore 85A) as the soft printing material. The detailed parameters of printing can be found in Table S2 (Supporting Information).

### Experiment Instruments and Software Platform

The output signals of the T‐TENG, L‐TENG, and PVDF sensor for sensor characterization were collected by an oscilloscope (DSOX3034A, Agilent) with 100 MΩ impedance. The output signals for the data set collection and IoT based applications were directly collected by the IoT module (Arduino Mega 2560) with a customized circuit for signal processing. For the soft robot actuation, the air pressure was detected by an air pressure sensor (ISE30A, SMC) and controlled by a reducing valve and solenoid valve. The ML‐based data analysis was conducted in the programming environment of Python and the network architecture was constructed with the help of Keras deep learning module.

## Conflict of Interest

The authors declare no conflict of interest.

## Supporting information

Supporting Information

Supplemental Movie 1

Supplemental Movie 2

Supplemental Movie 3

## Data Availability

The data that support the findings of this study are available from the corresponding author upon reasonable request.
