# Supplementary material for: Artificial Intelligence of Things (AIoT) Enabled Virtual Shop Applications Using Self‐Powered Sensor Enhanced Soft Robotic Manipulator
Source: Adv Sci (Weinh). 2021 May 26;8(14):2100230. doi: 10.1002/advs.202100230 (PMC8292889; doi:10.1002/advs.202100230)
Supplement: Supplementary file 1 — Supporting Information [file ADVS-8-2100230-s001.pdf]

WILEY-VCH

© 2021 Wiley-VCH GmbH

ADVANCED SCIENCE Open Access

Supporting Information

for *Adv. Sci.*, DOI: 10.1002/advs.202100230

Artificial Intelligence of Things (AIoT) Enabled Virtual Shop Applications Using Self-powered Sensor Enhanced Soft Robotic Manipulator

*Zhongda Sun, Minglu Zhu, Zixuan Zhang, Zhaocong Chen, Qiongfeng Shi, Xuechuan Shan, Raye Chen Hua Yeow and Chengkuo Lee**

© 2021 Wiley-VCH GmbH

Supporting Information

Artificial Intelligence of Things (AIoT) Enabled Virtual Shop Applications Using Self-powered Sensor Enhanced Soft Robotic Manipulator

*Zhongda Sun, Minglu Zhu, Zixuan Zhang, Zhaocong Chen, Qiongfeng Shi, Xuechuan Shan, Raye Chen Hua Yeow and Chengkuo Lee**

The file includes:

Figure S1. The structure and the working mechanism of the pneumatic actuator.

Figure S2. Analysis of the outputs generated in the T-TENG sensor for contact position and conact area detection.

Figure S3. Working mechanism of piezoelectric under compressive/tensile stress.

Figure S4. The set up to test the temperature sensing performance of the PVDF film.

Figure S5. Response time of the pyroelectric output generated in the PVDF sensor when the temperature of the contact object is 20 ℃, 25 ℃ and 30 ℃ respectively.

Figure S6. The output signal spectrums and the corresponding t-SNE visualization results for grasping elongated objects under different directions.

Figure S7. The confusion map for the recognition result of the 5 elongated objects.

Figure S8. The confusion map for the recognition result of the 28 different items with various shapes and sizes.

Figure S9. Illustration of the PVDF sensor integrated smart manipulator.

Figure S10. The confusion map for the recognition result based on 10-channel data set.

Figure S11. The confusion map for the recognition result of 28 items with the 5-channel data extracted from the original 15-channel data set.

Figure S12. Output performance of the PVDF sensor when integrated on the soft manipulator.

Figure S13. The confusion map for the recognition result of apple, orange, big box, long can and short can (labeled as 1-5) when one finger integrated with the TENG sensors with the number of short electrodes of (a) three and (b) four respectively.

Figure S14. Humidity effects on the T-TENG sensor output.

Table S1. Comparison of flexible solutions for soft robot perception.

Table S2. The technical parameters of 3D printing.

Table S3. The detailed parameters of the 1D-CNN ML architecture.

Table S4. The stability test of the ML-enabled integrated sensory system after cycles of utilization.

Table S5. The stability test of the ML-enabled integrated sensory system when grasping objects under different temperatures.

Supplementary Note 1. The current limit of the resolution for the TENG sensors and the influence of the resolution on the machine learning result.

Supplementary Note 2. Humidity effects on the T-TENG sensor output.

Other Supplementary Material for this manuscript includes the following:

Movie S1. Online virtual shop application.

Movie S2. Shape-size-temperature fused sensory system.

Movie S3. User interface for temperature distribution monitoring.

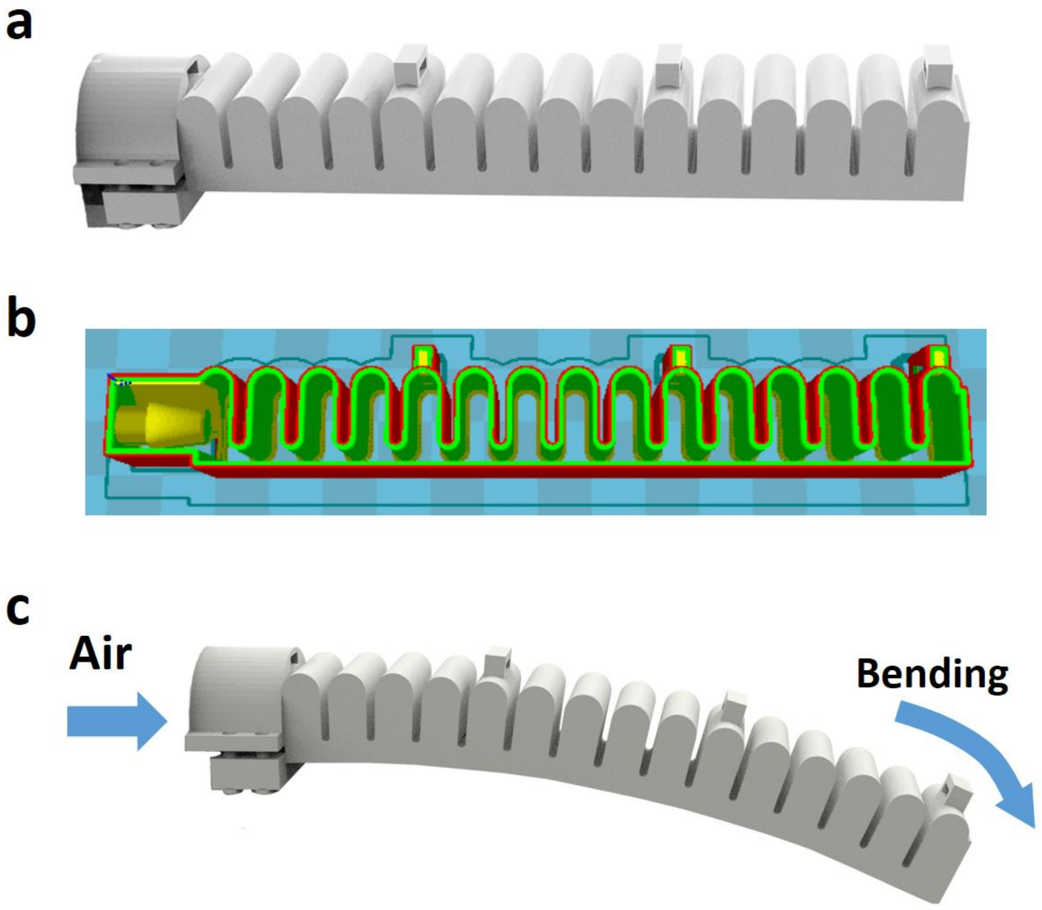

Figure S1. The structure and the working mechanism of the pneumatic actuator. (a) Illustration of the detailed hollow-bellows structure of the pneumatic actuator. (b) The corresponding cross-section figure. (c) Bending down motion of the pneumatic actuator when applied air pressure.

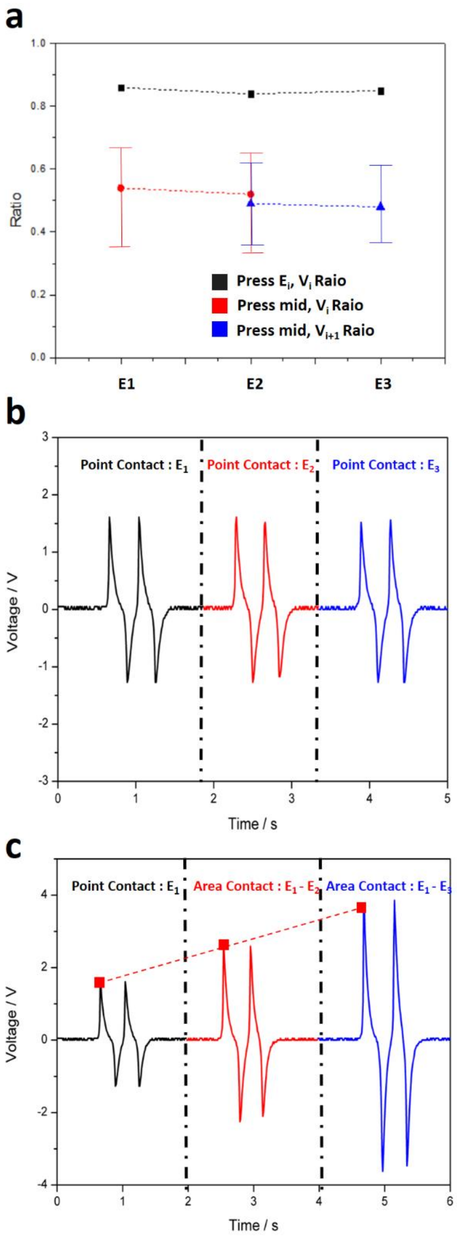

Figure S2. Analysis of the outputs generated in the T-TENG sensor for contact position and conact area detecting. (a) Voltage ratio analysis of the generated ouputs in T-TENG short electrodes (E_1-E_3) by tapping different positions of the T-TENG sensor patch. (b) Outpus generated in the long electrode (E_L) by tapping different positions with the same area. (c) Outputs generated in E_L by tapping with different areas.

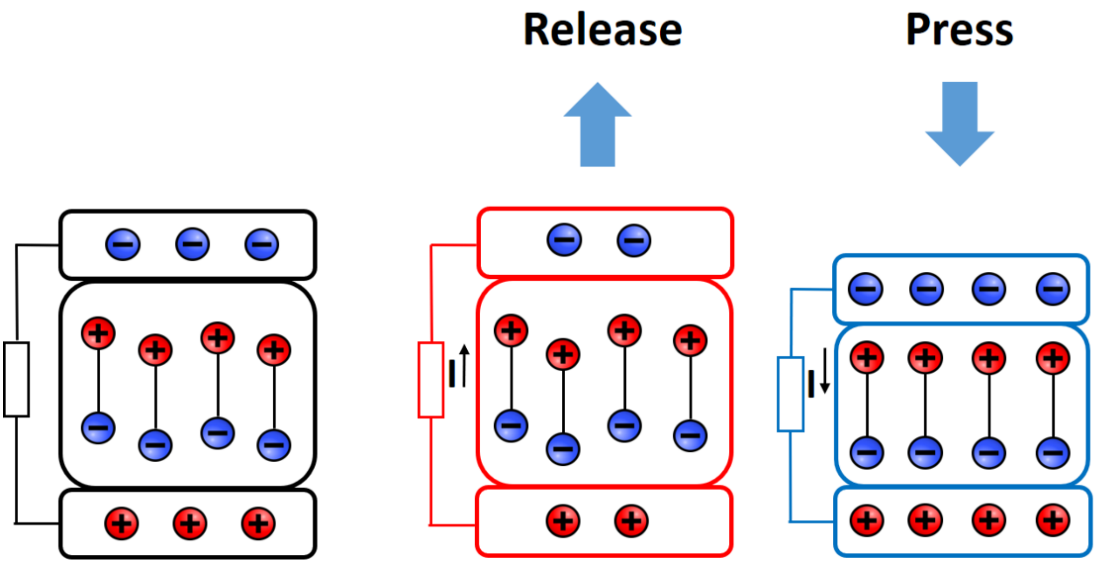

Figure S3. Working mechanism of piezoelectric under compressive/tensile stress.

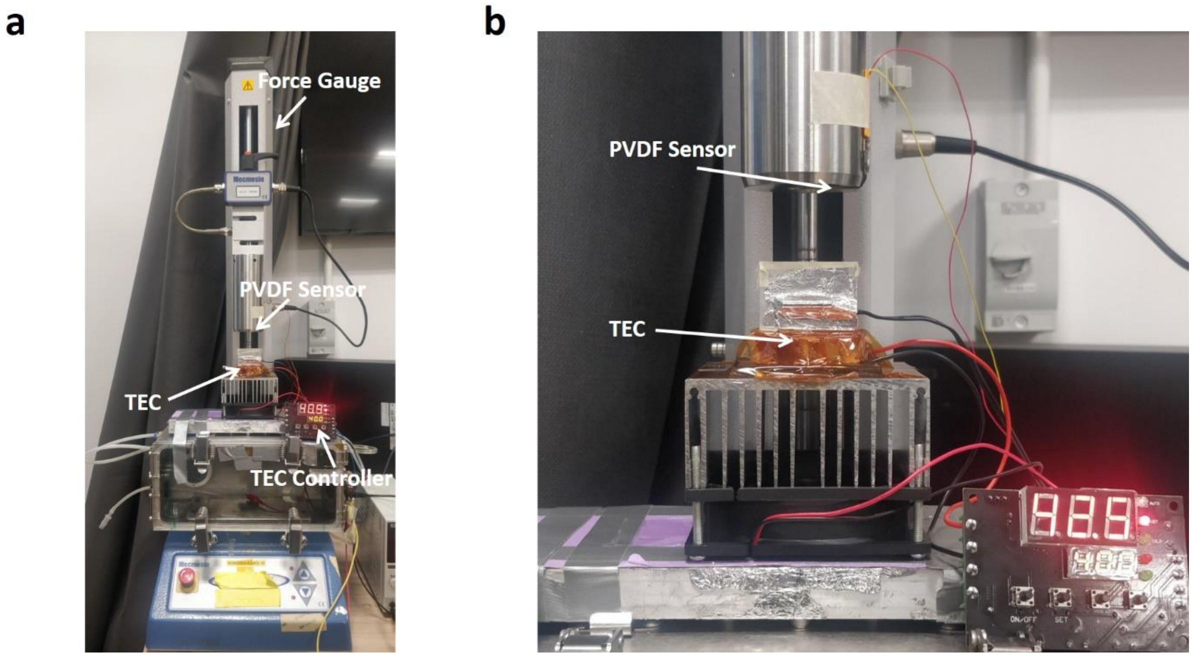

Figure S4. The set up to test the temperature sensing performance of the PVDF film. (a) Illustraion of a force gauge to control the contact force, and a TEC as the contact object with a TEC controller to control the temperature. (b) Enlarged figure shows the position where the PVDF sensor attached to the moving load.

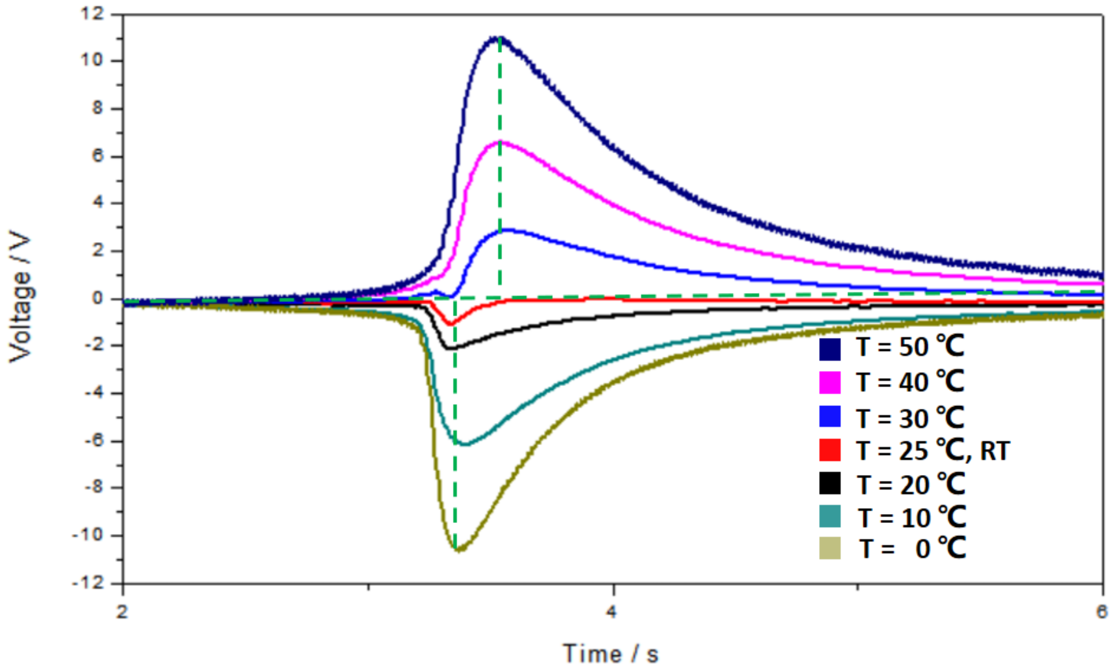

Figure S5. Response time of the pyroelectric output generated in the PVDF sensor when the temperature of the contact object is 0℃, 10℃, 20 ℃, 25 ℃, 30 ℃, 40℃ and 50 ℃ respectively.

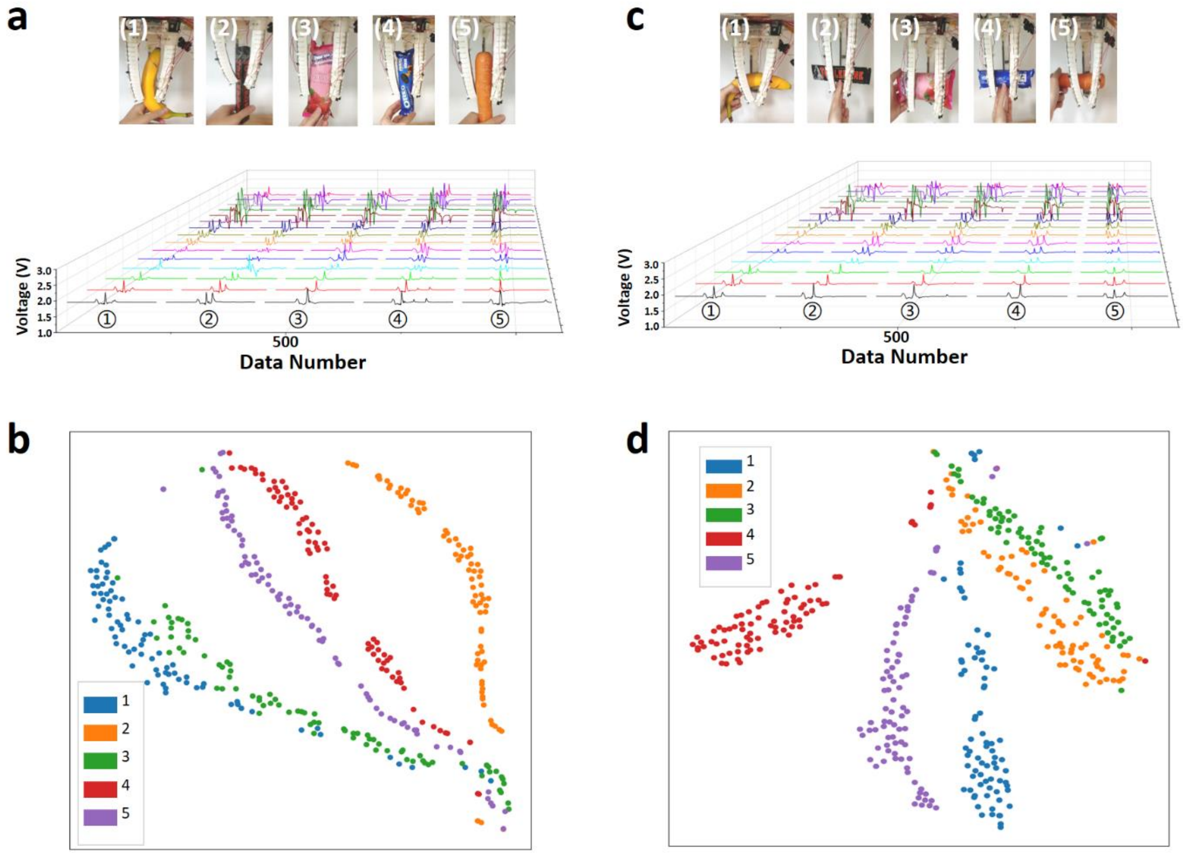

Figure S6. The output signal spectrums and the corresponding t-SNE visualization results for grasping elongated objects under different directions. (a) The typical 15-channel output spectra collected by the integrated TENG sensing system and (b) the corresponding t-SNE visualization results of the 5 elongated objects grasped by the manipulator vertically. (c) The typical 15-channel output spectrums collected by the integrated TENG sensing system and (d) the corresponding t-SNE visualization results of the 5 elongated objects grasped by the manipulator horizontally.

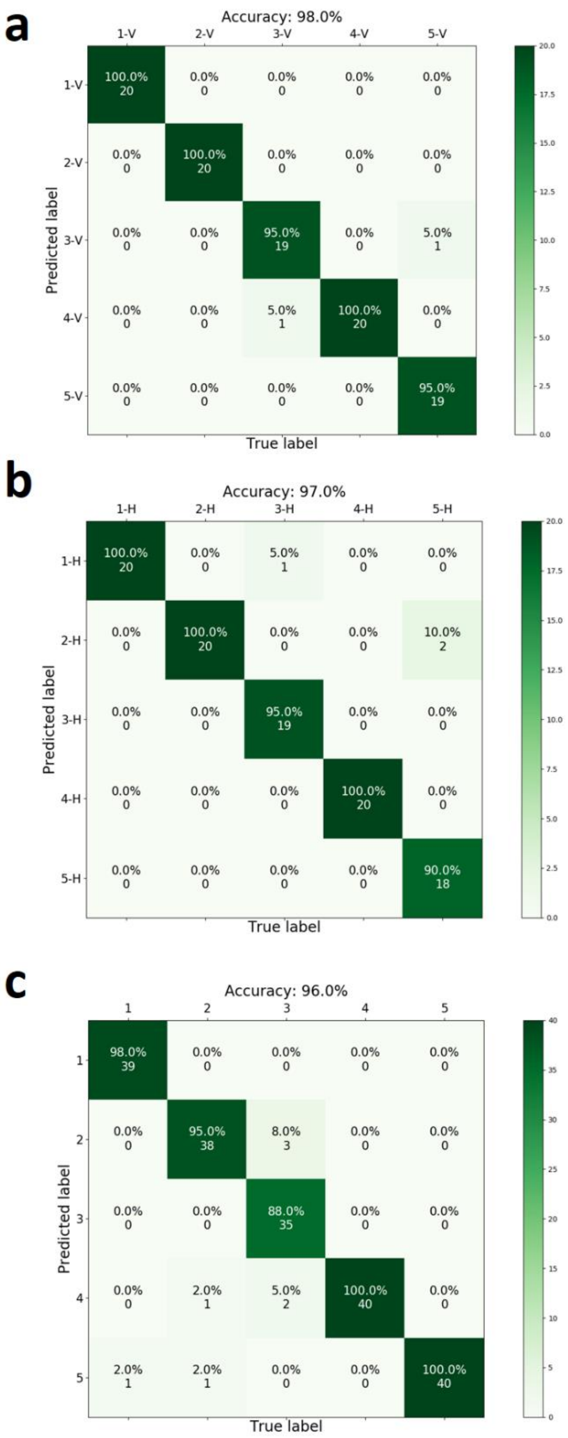

Figure S7. The confusion map for the recognition result of the 5 elongated objects grasped (a) vertically, (b) horizontally. (c) The confusion map for the recognition result of the 5 elongated objects when the data of grasping vertically and horizontally are fused toghther.

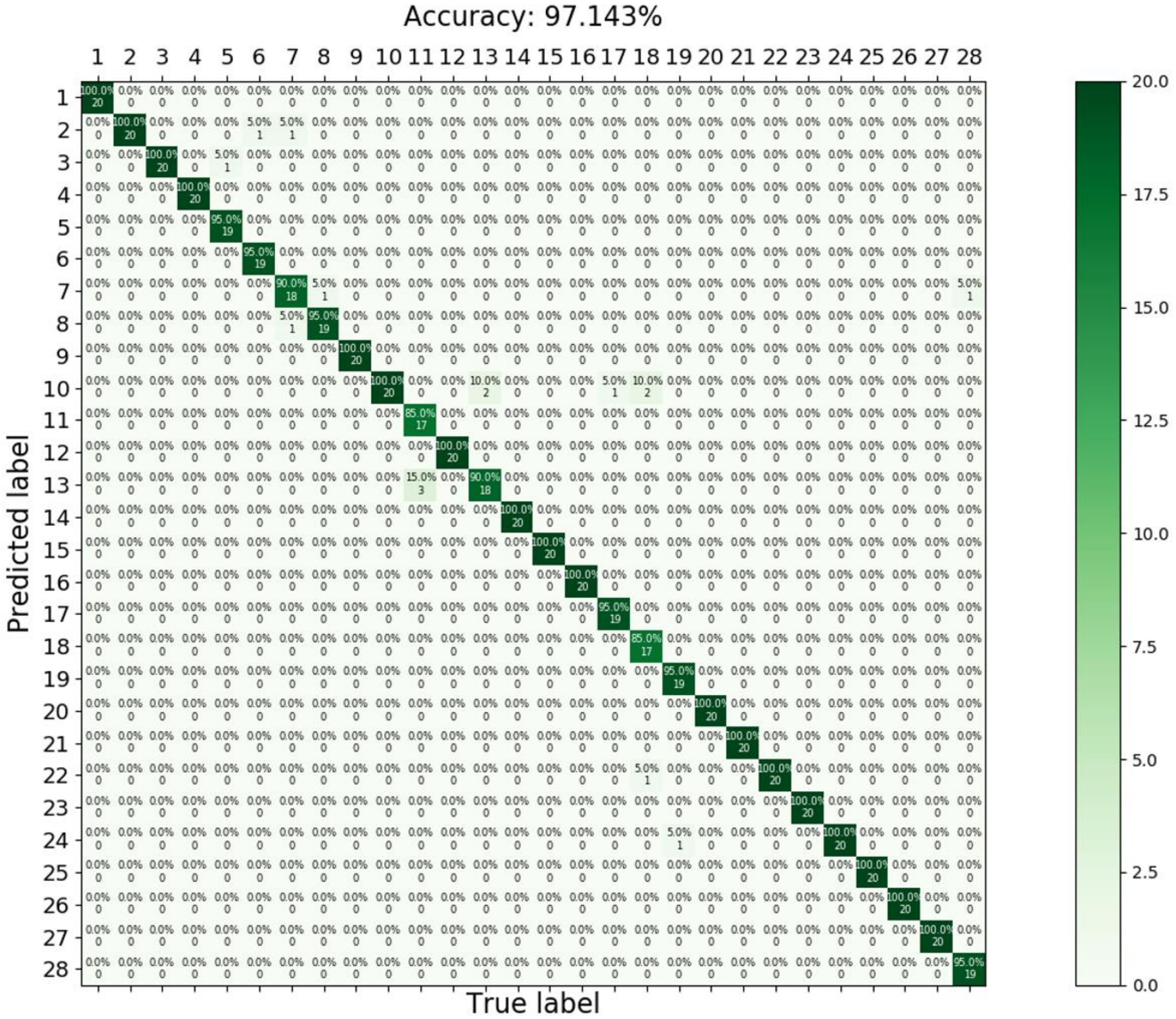

Figure S8. The confusion map for the recognition result of the 28 different items with various shapes and sizes.

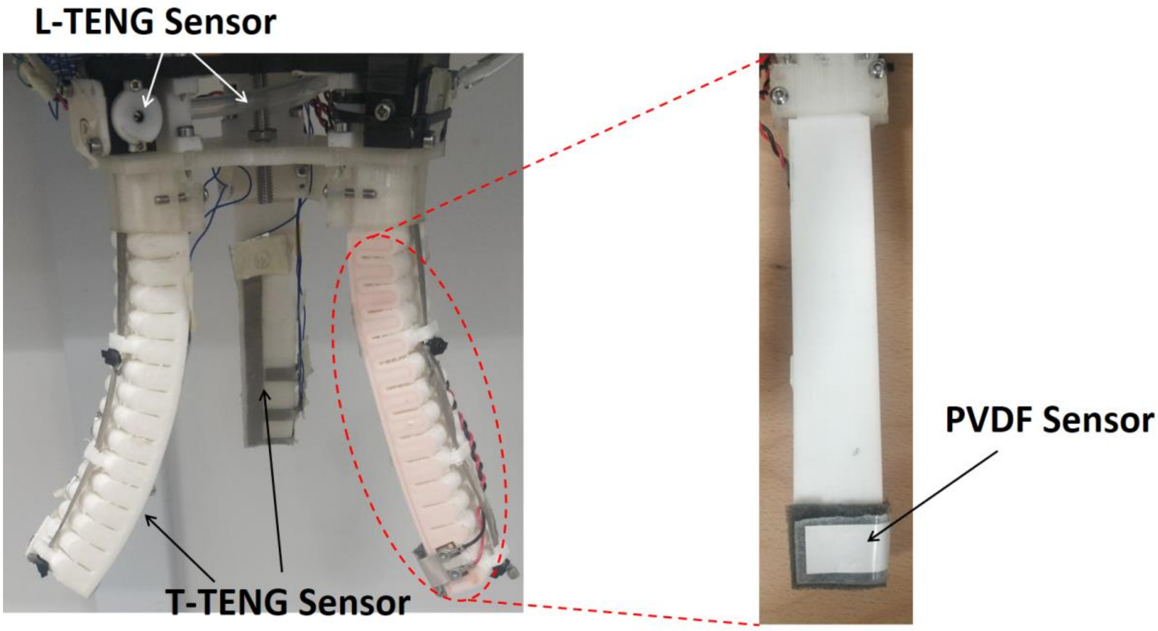

Figure S9. Illustration of the PVDF sensor integrated smart manipulator

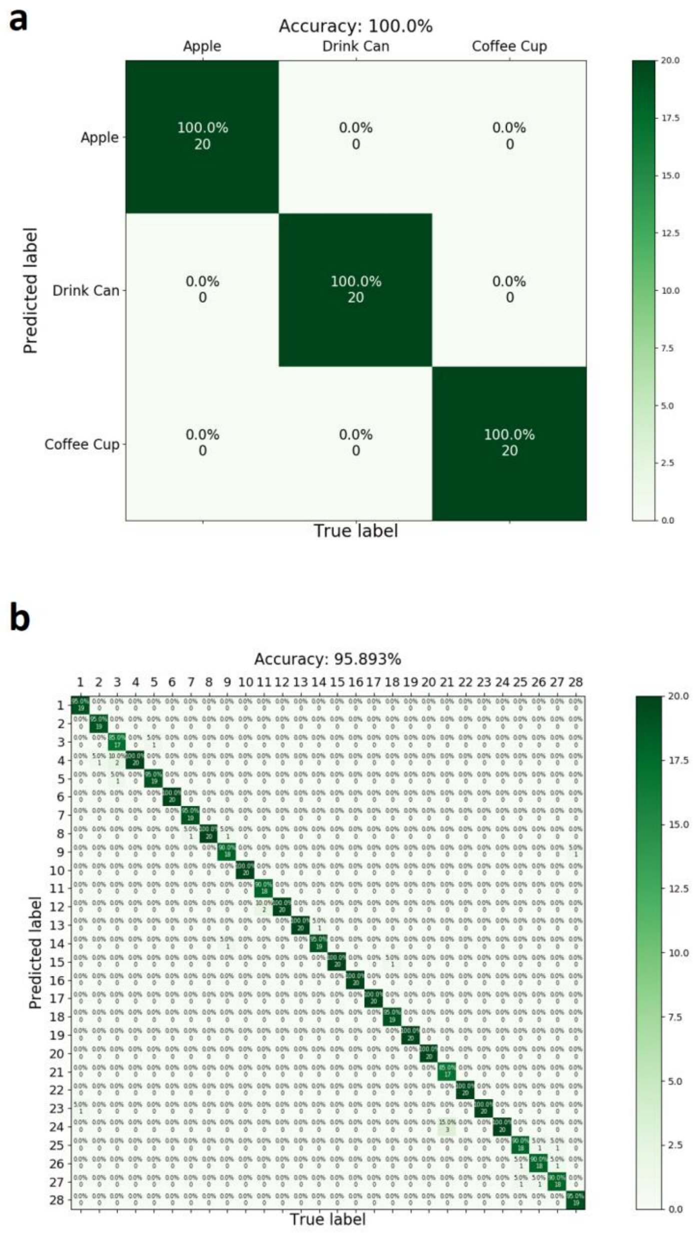

Figure S10. The confusion map for the recognition result based on 10-channel data set. (a) The confusion map for the recognition result of apple, drink can and coffee cup with the 10-channel data collected by the TENG sensors integrated on two pneumatic fingers. (b) The confusion map for the recognition result of 28 items with the 10-channel data extracted from the original 15-channel data set.

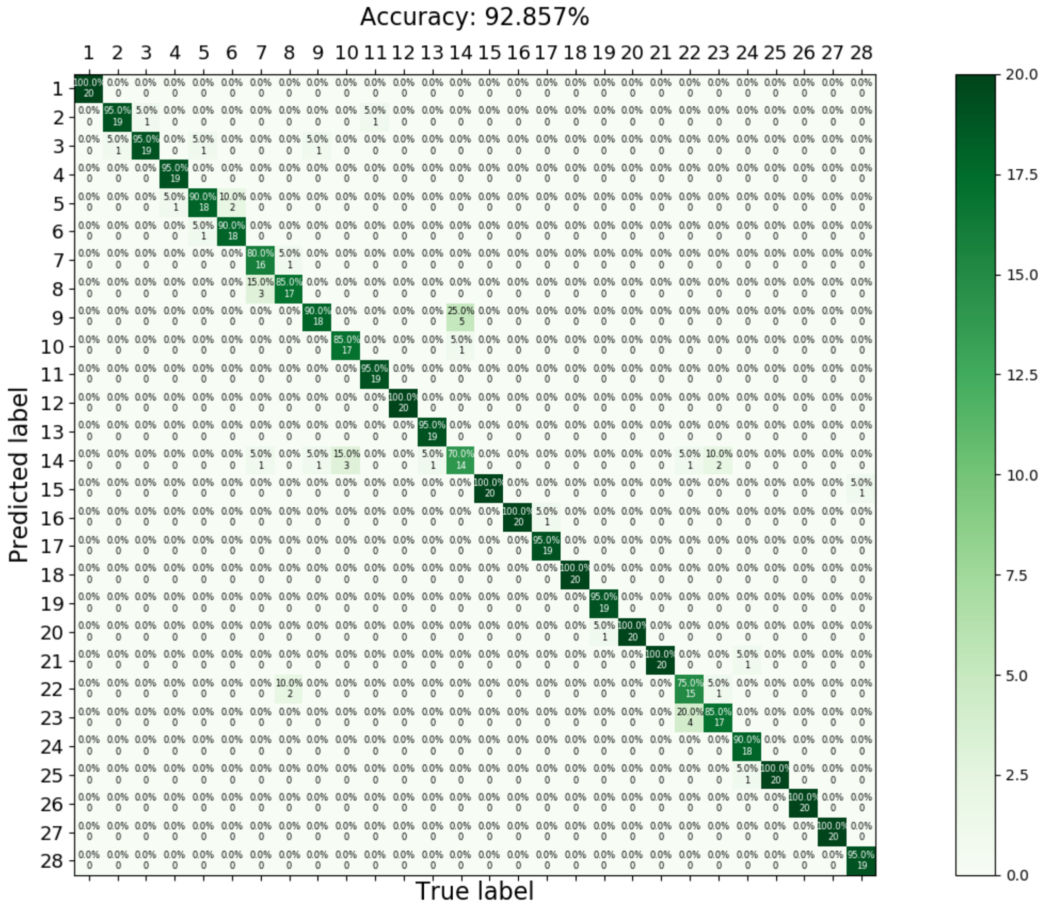

Figure S11. The confusion map for the recognition result of 28 items with the 5-channel data extracted from the original 15-channel data set.

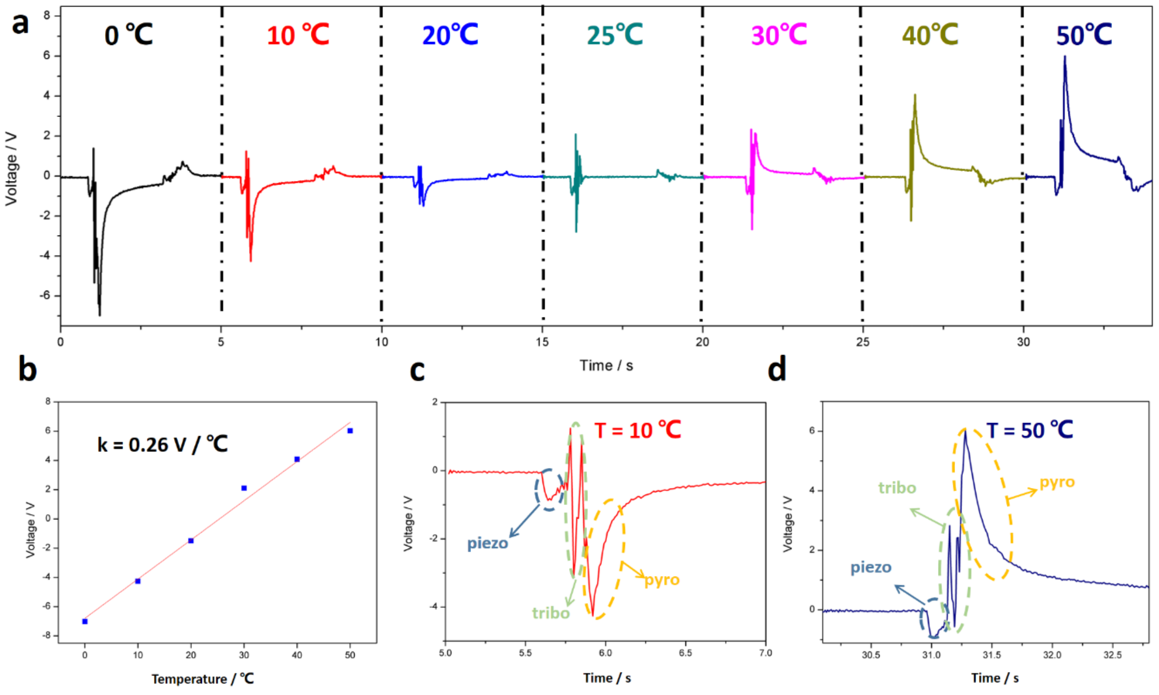

Figure S12. Output performance of the PVDF sensor when integrated on the soft manipulator. (a) The piezoelectric-triboelectric-pyroelectric superimposed output of the PVDF sensor when contact with TEC under different temperatures with the air pressure in the chamber of the pneumatic finger fixed at 130 kPa. (b) The calculated peak values of the PVDF sensor during contact motion when the temperature of the TEC equals to 0℃, 10℃, 20℃, 30℃, 40℃ and 50℃. The detailed output signal of the PVDF sensor when contact with TEC at (c) 10℃ and (d) 50℃ respectively.

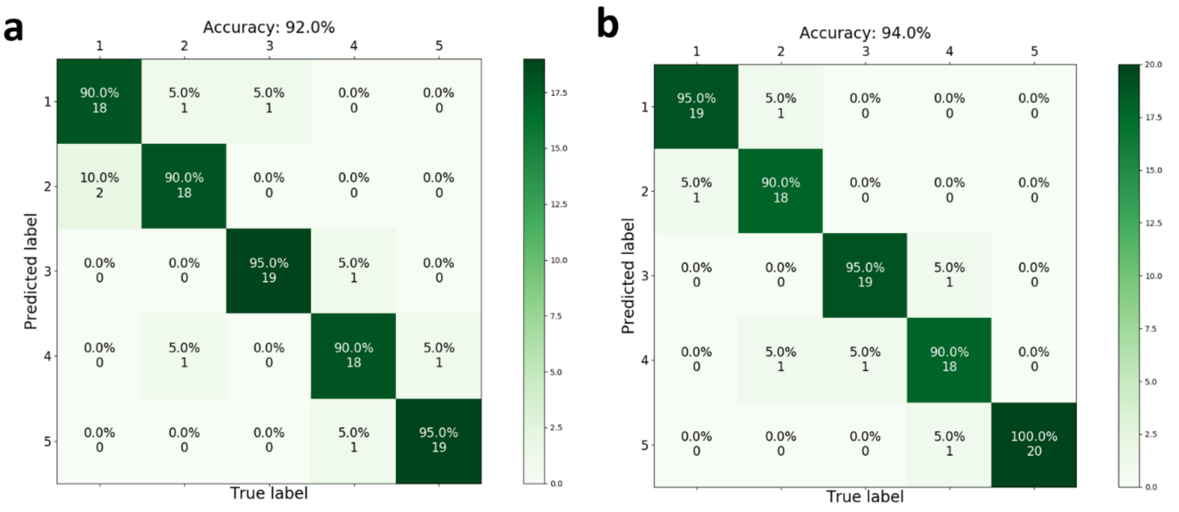

Figure S13. The confusion map for the recognition result of apple, orange, big box, long can and short can (labeled as 1-5) when one finger integrated with the TENG sensors with the number of short electrodes of (a) three and (b) four respectively.

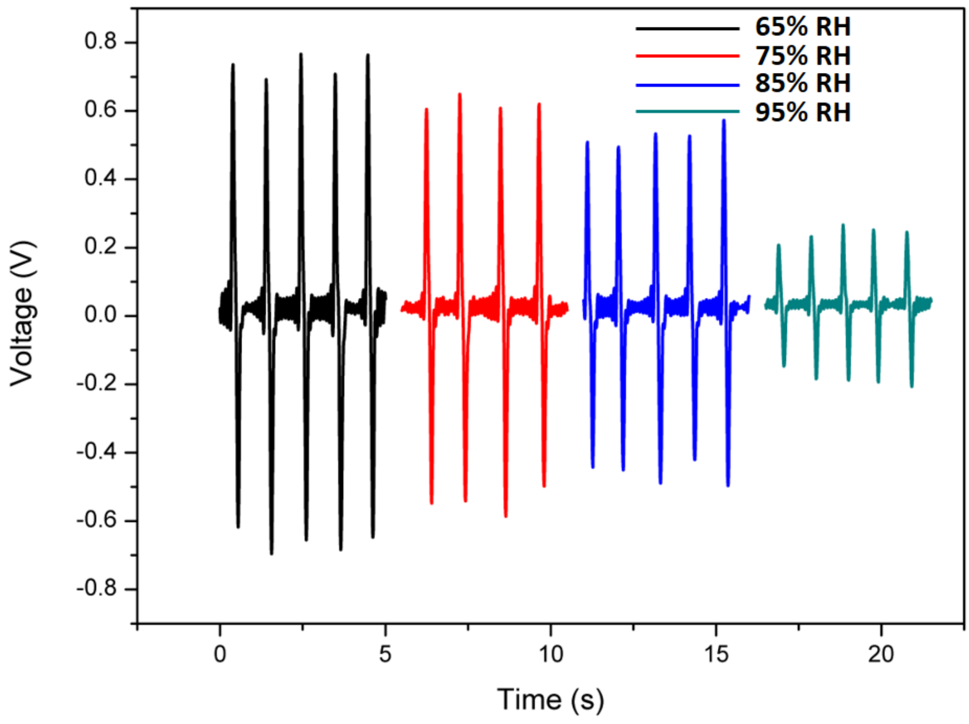

Figure S14. Humidity effects on the T-TENG sensor output.

Table S1. Comparison of flexible solutions for soft robot perception.

**Comparison of flexible solutions for soft robot perception**					
Mechanism	Material	Tactile	Deformation	Temperature	Self-powered
Microfluidic[1]	PDMS Channel	Yes	Yes	No	No
Capacitive[2]	Galinstan	No	Yes	No	No
Resistive[3]	PLA-CNT	No	Yes	No	No
Optical fibers[4]	Illuminated elastomer foam	No	Yes	No	No
Resistive[5]	CNT	Yes	Yes	No	No
Resistive[6]	cPDMS	No	Yes	No	No
Optical waveguides[7]	LED, photodiode, elastomer	Yes	Yes	No	No
Piezoelectric[8]	PVDF	No	Yes	No	Yes
Triboelectric[9]	Ecoflex	Yes	No	No	Yes
Triboelectric[10]	PDMS	No	Yes	No	Yes
Triboelectric & Potentiometric[11]	PDMS & Ionic composite electrolyte	Yes	No	No	Yes
Triboelectric[12,13]	Ecofiex & PTFE	Yes	Yes	No	Yes
Triboelectric[14]	Silicone rubber	No	Yes	No	Yes
Piezoresistive & Pyroelectric[15]	Graphite-CNT & PVDF	Yes	Yes	Yes	Partly

Table S2. The technical parameters of 3D printing.

**Quality**	
Layer height (mm)	0.1
Shell thickness (mm)	1.6
Enable retraction	☐
**Fill**	
Bottom/Top thickness (mm)	2
Fill Density (%)	0
**Speed and Temperature**	
Print speed (mm/s)	30
Printing temperature (C)	230
Bed temperature (C)	0
**Support**	
Support type	None
Platform adhesion type	None
**Filament**	
Diameter (mm)	1.5
Flow (%)	100.0
**Machine**	
Nozzle size (mm)	0.4

**Retraction**	
Speed (mm/s)	60
Distance (mm)	8
**Quality**	
Initial layer thickness (mm)	0.15
Initial layer line width (%)	140
Cut off object bottom (mm)	0.0
Dual extrusion overlap (mm)	0.2
**Speed**	
Travel speed (mm/s)	120
Bottom layer speed (mm/s)	30
Infill speed (mm/s)	30
Top/bottom speed (mm/s)	30
Outer shell speed (mm/s)	30
Inner shell speed (mm/s)	30
**Cool**	
Minimal layer time (sec)	5
Enable cooling fan	☑

Table S3. The detailed parameters of the 1D-CNN ML architecture.

No	Layer	No. of Filters	Kernel/ Pool Size	Stride	Input Size	Output Size	Padding
1	Convolution 1	32	5	1	(None,200,15)	(None,200,32)	Same
2	Max-pooling 1		2	2	(None,200,32)	(None,100,32)	Same
3	Convolution 2	64	5	1	(None,100,32)	(None,100,64)	Same
4	Max-pooling 2		2	2	(None,100,64)	(None,50,64)	Same
5	Convolution 3	128	5	1	(None,50,64)	(None,50,128)	Same
6	Max-pooling 3		2	2	(None,50,128)	(None,25,128)	Same
7	Flatten				(None,25,128)	(None,3200)	
8	Dense(3000)				(None,3200)	(None,3000)	
9	Dense(28)				(None,3000)	(None,28)	

Table S4. The stability test of the ML-enabled integrated sensory system after cycles of utilization.

	Cycles of Utilization			
	500	1000	1500	2000
	Correct / Total	Correct / Total	Correct / Total	Correct / Total
Baseball	19/20	18/20	17/20	18/20
Egg	20/20	19/20	18/20	19/20
Big box	20/20	20/20	20/20	19/20
Cup	19/20	20/20	20/20	20/20
Tape	19/20	19/20	20/20	20/20
Accuracy	97/100 (97%)	96/100 (96%)	95/100 (95%)	96/100 (96%)

Note: “Correct/Total” means the correct times in total numbers testing.

Table S5. The stability test of the ML-enabled integrated sensory system when grasping objects under different temperatures.

	Object Temperature				
	0 ℃	10 ℃	25 ℃	40 ℃	50 ℃
	Correct / Total	Correct / Total	Correct / Total	Correct / Total	Correct / Total
Coffee Cup		20/20 （100%）	19/20 （95%）	19/20 （95%）	20/20 （100%）
Pepsi Can	19/20 （95%）	20/20 （100%）	20/20 （100%）		

Note: “Correct/Total” means the correct times in total numbers testing.

Supplementary Notes

Supplementary Note 1. The current limit of the resolution for the TENG sensors and the influence of the resolution on the machine learning result.

The size of the L-TENG and T-TENG sensors is determined by the available space with a certain design or size of the manipulator structure. For the L-TENG sensor, the larger the size of the manipulator structure or more reserved space for the sensor, the larger gear that can be installed, thus reserving more space on the gear surface for more gear teeth and an improved detecting resolution due to the greater number of teeth under a certain arc of rotation. Besides, with a specific size of the L-TENG sensor or the gear, we can further utilize advanced fabrication processes, such as MEMS process, micro-machining, etc., to increase the teeth density and improve the resolution. Considering the relatively small size of the current manipulator design and the low cost of the 3D-printing fabrication process, the current resolution of the L-TENG sensor is acceptable as one feature of the object size for further machine learning. For the T-TENG sensor, the resolution is determined by the gap between adjacent electrodes and the total number of electrodes within a certain area of the tactile panel. Commonly, for such a grating structure, more grating electrodes with a smaller gap will result in a higher resolution, and many high-resolution grating solutions based on triboelectric have been proposed before.[16,17] However, in our design, more grating electrodes also mean more channels of the output signal and more complex data to be used for machine learning. Though more features and information can be extracted to increase the recognition accuracy due to the increasing channel numbers, when the accuracy has reached a certain level, the cost and benefit will not be proportional. In our case, the 10 mm level resolution of the T-TENG sensors already

able to contribute a high accuracy of 97.143% when fused with the L-TENG sensor data, which is acceptable for the low cost and energy-saving purpose with a minimalistic design.

Additional test have been added to see the influence of the sensor resolution on the machine result. Due to the current limitation of the 3D-printing fabrication process we used and the specific manipulator size, the resolution of the L-TENG sensor is relatively difficult to be improved at this stage. However, it is easy for us to increase the number of short electrodes from three to four in the T-TENG sensors to improve the resolution from 10 mm to ~6.67 mm, and see the machine learning result. In the experiment, we integrated one pneumatic finger with the L-TENG sensor and the T-TENG sensor and test its recognition performance on 5 objects: apple, orange, big box, long can, and short can. Figure S13(a) shows that when the number of the short electrodes is three, the classification accuracy can reach 92%, and can be further improved to 94% when the number of the short electrodes increases to four depicted in Figure S13(b), proving that the enhanced resolution and the increased data/channel number will benefit the machine learning result.

Supplementary Note 2. Humidity effects on the T-TENG sensor output.

The main structure of the L-TENG sensor is packaged with the PLA material as shown in Figure 1(a-i), while the T-TENG sensor is directly exposed to the surrounding air and the contact surface of the objects. So the moisture in the air or on the surface of the object will have a greater impact on the outputs of the T-TENG sensor. Actually, the contact position detecting mechanism of the T-TENG sensor is based on the voltage ratio between the short electrodes.

Although the output of triboelectricity will become smaller under high humidity, the degree of change of all short electrodes' outputs is the same, so the ratio of each other will not change, and the position detection function will not be affected. However, for the long electrode which detects the contact area based on the output voltage amplitude, the detecting ability will be influenced.

To further evaluate the humidity effects on the T-TENG sensor output, we have done the extra experiments under different humidity conditions as shown in Figure S14. The normal relative humidity in the surrounding air is about 70% - 80% RH. It is clear that the output voltage amplitude of the T-TENG sensor declines gradually when the humidity increase from 65% to 85%, and drops sharply when the relative humidity reaches 95%. Because we use the voltage spectrum generated during the process of grasping objects directly for machine learning, the variation in voltage amplitude under different humidity will inevitably affect the results of machine learning. However, if we collect the data under different humidity and combine them into a more generalized data set, i.e. the category of each object contains the data that the object was captured under different humidity conditions, the influence of humidity on accuracy will be avoided to a certain extent.

Supplementary References

[1] A. Konda, D. Lee, T. You, X. Wang, S. Ryu, S. A. Morin, *Adv. Intell. Syst.* **2019**, *1*, 1900027.

[2] L. Y. Zhou, Q. Gao, J. F. Zhan, C. Q. Xie, J. Z. Fu, Y. He, *ACS Appl. Mater. Interfaces* **2018**, *10*, 23208.

[3] S. Mousavi, D. Howard, F. Zhang, J. Leng, C. H. Wang, *ACS Appl. Mater. Interfaces* **2020**, *12*, 15631.

[4] I. M. Van Meerbeek, C. M. De Sa, R. F. Shepherd, *Sci. Robot.* **2018**, *3*, 1.

[5] R. Goldoni, Y. Ozkan-Aydin, Y. S. Kim, J. Kim, N. Zavanelli, M. Mahmood, B. Liu, F. L. Hammond, D. I. Goldman, W. H. Yeo, *ACS Appl. Mater. Interfaces* **2020**, *12*, 43388.

[6] T. G. Thuruthel, B. Shih, C. Laschi, M. T. Tolley, *Sci. Robot.* **2019**, *4*.

[7] H. Zhao, K. O'Brien, S. Li, R. F. Shepherd, *Sci. Robot.* **2016**, *1*, DOI 10.1126/scirobotics.aai7529.

[8] M. Xie, M. Zhu, Z. Yang, S. Okada, S. Kawamura, *Nano Energy* **2021**, *79*, 105438.

[9] Y. C. Lai, J. Deng, R. Liu, Y. C. Hsiao, S. L. Zhang, W. Peng, H. M. Wu, X. Wang, Z. L. Wang, *Adv. Mater.* **2018**, *30*, 1.

[10] M. Zhu, M. Xie, X. Lu, S. Okada, S. Kawamura, *Nano Energy* **2020**, *73*, 104772.

[11] X. Wu, J. Zhu, J. W. Evans, A. C. Arias, *Adv. Mater.* **2020**, *2005970*, 1.

[12] S. Chen, Y. Pang, H. Yuan, X. Tan, C. Cao, *Adv. Mater. Technol.* **2020**, *5*, 1.

[13] T. Jin, Z. Sun, L. Li, Q. Zhang, M. Zhu, Z. Zhang, G. Yuan, T. Chen, Y. Tian, X. Hou, C. Lee, *Nat. Commun.* **2020**, *11*, 1.

[14] J. Chen, K. Han, J. Luo, L. Xu, W. Tang, Z. L. Wang, *Nano Energy* **2020**, *77*, 105171.

[15] X. Q. Wang, K. H. Chan, Y. Cheng, T. Ding, T. Li, S. Achavananthadith, S. Ahmet, J. S. Ho, G. W. Ho, *Adv. Mater.* **2020**, *32*, 1.

[16] Z. Wang, J. An, J. Nie, J. Luo, J. Shao, T. Jiang, B. Chen, W. Tang, Z. L. Wang, *Adv. Mater.* **2020**, *32*, 1.

[17] Q. Jing, Y. S. Choi, M. Smith, N. Ćatić, C. Ou, S. Kar-Narayan, *Adv. Mater. Technol.* **2019**, *4*, 1.
